# Therapeutic Potential of Targeting the SUMO Pathway in Cancer

**DOI:** 10.3390/cancers13174402

**Published:** 2021-08-31

**Authors:** Antti Kukkula, Veera K. Ojala, Lourdes M. Mendez, Lea Sistonen, Klaus Elenius, Maria Sundvall

**Affiliations:** 1 Cancer Research Unit, FICAN West Cancer Center Laboratory, Institute of Biomedicine, Turku University Hospital, University of Turku, FI-20520 Turku, Finland; antti.v.kukkula@utu.fi (A.K.); vekaoj@utu.fi (V.K.O.); klaele@utu.fi (K.E.); 2 Turku Doctoral Programme of Molecular Medicine, University of Turku, FI-20520 Turku, Finland; 3 Medicity Research Laboratories, University of Turku, FI-20520 Turku, Finland; 4 Turku Bioscience Centre, University of Turku and Åbo Akademi University, FI-20520 Turku, Finland; lea.sistonen@abo.fi; 5 Beth Israel Deaconess Cancer Center, Beth Israel Deaconess Medical Center, Department of Medicine and Pathology, Cancer Research Institute, Harvard Medical School, Boston, MA 02115, USA; lmendez@bidmc.harvard.edu; 6 Faculty of Science and Engineering, Cell Biology, Åbo Akademi University, FI-20520 Turku, Finland; 7 Department of Oncology, Turku University Hospital, FI-20521 Turku, Finland

**Keywords:** cancer, post-translational modification (PTM), small ubiquitin-like modifier (SUMO), protein inhibitor of activated STAT (PIAS), sentrin-specific protease (SENP)

## Abstract

**Simple Summary:**

The small ubiquitin-like modifier (SUMO) pathway regulates the hallmark properties of cancer cells. Moreover, alterations in activity and in levels of SUMO machinery components have been observed in human cancer. Due to the reversible nature of this post-translational protein modification, the balance between SUMOylation and the removal of SUMO is critical. Early-phase clinical trials are currently evaluating the safety and efficacy of SUMO pathway inhibition in cancer patients. In this comprehensive review, we critically discuss the potential of targeting the SUMO pathway as a therapeutic option for cancer.

**Abstract:**

SUMOylation is a dynamic and reversible post-translational modification, characterized more than 20 years ago, that regulates protein function at multiple levels. Key oncoproteins and tumor suppressors are SUMO substrates. In addition to alterations in SUMO pathway activity due to conditions typically present in cancer, such as hypoxia, the SUMO machinery components are deregulated at the genomic level in cancer. The delicate balance between SUMOylation and deSUMOylation is regulated by SENP enzymes possessing SUMO-deconjugation activity. Dysregulation of SUMO machinery components can disrupt the balance of SUMOylation, contributing to the tumorigenesis and drug resistance of various cancers in a context-dependent manner. Many molecular mechanisms relevant to the pathogenesis of specific cancers involve SUMO, highlighting the potential relevance of SUMO machinery components as therapeutic targets. Recent advances in the development of inhibitors targeting SUMOylation and deSUMOylation permit evaluation of the therapeutic potential of targeting the SUMO pathway in cancer. Finally, the first drug inhibiting SUMO pathway, TAK-981, is currently also being evaluated in clinical trials in cancer patients. Intriguingly, the inhibition of SUMOylation may also have the potential to activate the anti-tumor immune response. Here, we comprehensively and systematically review the recent developments in understanding the role of SUMOylation in cancer and specifically focus on elaborating the scientific rationale of targeting the SUMO pathway in different cancers.

## 1. Introduction

Cancer is consistently ranked among the leading causes of death worldwide [1]. Hallmarks of cancer cells include uncontrolled proliferation, inhibition of apoptosis and differentiation, immune evasion, and the potential to metastasize [2]. Cellular homeostasis and responses to various stimuli are regulated by a wide array of post-translational modifications (PTM), including phosphorylation, acetylation, hydroxylation, glycosylation, and methylation as well as small protein modifications, such as ubiquitination [3,4]. Dysregulation of PTMs is frequent in cancer and plays a significant role in the pathogenesis of the disease [5,6]. Ubiquitination and its analogous modifications neddylation and SUMOylation are increasingly viewed as key regulators of tumorigenesis of various cancers [7,8,9]. In this review, we discuss SUMOylation’s contribution to the malignant phenotype and the pathway’s role as a potential therapeutic target in cancers originating from different cell types and organs.

## 2. Basic Principles of SUMOylation and Its Role in Physiology

Small ubiquitin-like modifiers (SUMOs) are a group of ubiquitin-like small proteins that are attached to substrate proteins in a reversible post-translational modification termed SUMOylation that is highly conserved in eukaryotes [9,10]. The SUMO pathway is involved in several central cellular processes. Modification by SUMO mediates the protein–protein interactions of target substrates, and influences their subcellular localization, stability, and enzymatic function [9,11]. SUMOylation can simultaneously target several members of a protein group to modulate the activity of a specific pathway [12]. The SUMO pathway shares some structural and functional similarities with the ubiquitin pathway [11]. The global molecular structure of SUMO is similar to ubiquitin, and both have a ββαββαβ fold structure and a conserved position of a diglycine (GG) motif required for isopeptide bond formation [13,14].

**Figure 1:**
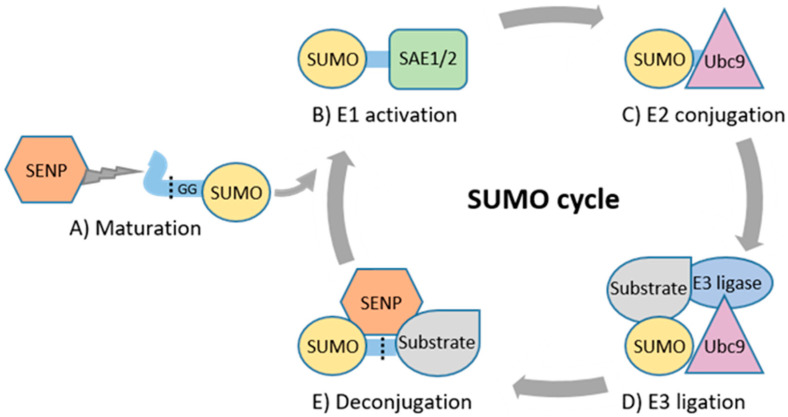
The enzymatic SUMO cascade. (**A**) SENPs cleave off amino acids from precursor SUMO to produce a mature SUMO. (**B**) SAE1/SAE2 forms of a thioester bond between SUMO’s GG and catalytic cysteine residue of SAE1/2. (**C**) Thioester bond is formed between SUMO’s GG and catalytic cysteine residue of Ubc9. (**D**) Ubc9 catalyzes formation of an isopeptide bond between GG and target substrate’s lysine residue often with assistance of an E3 ligase. (**E**) SENPs deconjugate SUMO from the substrate.

To date, five isoforms of SUMO (SUMO1, 2, 3, 4, and 5) have been identified in the human genome [9]. SUMO1, SUMO2, and SUMO3 are ubiquitously expressed in tissues, while the expression of SUMO4 and SUMO5 is restricted only to specific tissues [9,15,16,17,18]. SUMO1, SUMO2, and SUMO3 account for most of SUMO modifications, whereas the functional roles of SUMO4 and SUMO5 are more unclear [15,18]. SUMO2 and SUMO3 both share 97% sequence identity, but share only 50% sequence similarity with SUMO1 [19]. Expression levels of SUMOs are dynamic and fluctuate between different developmental stages [17]. SUMOs can be covalently attached to a single lysine residue (monoSUMOylation) or to multiple lysine residues (multiSUMOylation) of the substrate (Figure 1). SUMO is preferentially attached to SUMO consensus ΨKxE motifs of substrates, in which Ψ denotes a hydrophobic amino acid, K is lysine, x is any amino acid and E is glutamic acid, although some substrates are also SUMOylated at lysines in non-consensus sites [10,20,21]. SUMO itself may also be SUMOylated (polySUMOylation) to form polymeric SUMO chains, which are restricted to SUMO2 and SUMO3 [22]. SUMO1 does not form chains efficiently and is predominantly associated with monoSUMOylation [22]. However, SUMO1 can be attached to SUMO2/3 chains, resulting in the termination of chain elongation [23]. Non-covalent attachment of SUMO is regulated through the SUMO interaction motif (SIM) of binding partners [24,25].

The enzymatic cascade regulating SUMOylation comprises SUMO E1-activating, E2-conjugating, E3 ligase and deconjugating enzymes (Figure 1) [9]. Maturation and deconjugation (deSUMOylation) of SUMO is regulated by the family of cysteine proteases, sentrin-specific proteases (SENP1, 2, 3, 5, 6, 7, and 8) [26]. SENP1–7 have specific, varying subcellular localizations and regulate SUMO processing, whereas SENP8 displays substrate specificity only for ubiquitin-like protein NEDD8 [26,27,28]. SUMO E1-activating enzyme (SAE1/2) activates mature SUMO and transfers it to the only known mammalian E2-conjugating enzyme, Ubc9 (encoded by *UBE2I*) [29,30,31]. Ubc9 alone is capable of conjugating SUMO to substrates in vitro. However, E3 ligase enzymes are required for specificity and efficiency of SUMOylation. The family of protein inhibitors of activated STAT (PIAS) is the major class of SUMO E3 ligases, and the human genome has four PIAS genes (PIAS1, 2, 3, and 4) that encode seven protein products, PIAS1, PIAS2a (PIASxα), PIAS2b (PIASxβ), PIAS3, PIAS3L, PIAS4 (PIASy), and PIASyE6− [32,33]. Other classes of SUMO E3 ligases include the well-characterized Ran-binding protein 2 (RanBP2), and few other E3 ligases, such as ZNF451, polycomb protein PC2 (also known as CBX4), and some members of the tripartite motif (TRIM) protein family [34,35,36,37,38].

The SUMO pathway undergoes constant cross talk with other post-translational modifications, including phosphorylation and methylation [39,40,41]. For instance, phosphorylation can influence SUMOylation of substrates containing the conserved phosphorylation-dependent SUMOylation motif (PDSM), which mediates phosphorylation-dependent SUMOylation of various transcriptional regulators [39]. In addition, lysine methylation of the KKxE motif can create a motif resembling the SUMO consensus site in substrates containing the KKxE motif [40]. HMGA2 SUMOylation is dependent on its methylation at K66 and K67 of KKAE motif by methyltransferase SET7/9. The KKxE motif is present in several other proteins, including the polyhomeotic complex 1 (PHC1), which also requires methylation for its complete SUMOylation.

The importance of SUMOylation in physiology is apparent from early development. A functional SUMO pathway is essential for embryogenesis. Loss of Ubc9 leads to severe defects of chromosome condensation and segregation, as well as disruption of nuclear organization, resulting in death at early embryonic development in mice [42]. SUMO1 knockout mice are viable, as SUMO2/3 can compensate for most functions of SUMO1 [17,43]. Interestingly, SUMO3^−/−^ mice are also viable, but the knockout of SUMO2 leads to severe developmental defects and early death before adulthood, indicating that SUMO2 is essential for the development. Balance of SUMOylation and deSUMOylation is critical for embryonic development, as knockout of SENP1, SENP2, or SENP3 results in embryonic lethality in mice, suggesting non-redundancy and substrate specificity among SENPs [44,45,46]. SENP1^−/−^ mice have severe fetal anemia and SENP2^−/−^ mice display defects in trophoblast development, whereas conditional knockout Senp3^+/−^ mice have impaired cytokine and inflammatory signaling. PIAS1^−/−^ and PIAS4^−/−^ knockout mice are produced at a lower frequency than the expected Mendelian ratio due to increased perinatal lethality, and both knockouts display defects in cytokine signaling [47,48]. Surviving PIAS1^−/−^ mice are runts compared to their wild-type counterparts, whereas PIAS4^−/−^ mice show no obvious phenotype. PIAS2^−/−^ knockout mice have reduced testis weight and sperm count but remain viable and fertile [49]. PIAS3^−/−^ mice are viable and show no overt phenotype but have an impaired visual response of medium wavelength cones [50].

## 3. Altered Expression and Prognostic Significance of SUMO Pathway in Cancer

Dysregulated mRNA and protein levels of the SUMO machinery components have been reported in tissue samples of cancer patients with potential prognostic associations (Table S1 and Table 1). In the majority of the published reports, the expression levels of SUMO pathway components are upregulated in cancer and are associated with a higher histological grade, higher stage, presence of metastases and poor prognosis. Interestingly, genome-wide analyses have identified concurrently increased levels of multiple SUMO machinery components in certain cancer types [51]. However, high expression has been associated with better prognosis in some cancer types. Downregulated expression levels in cancer tissues have consistently been reported for SENP2 [52,53,54,55,56,57] and PIAS2 [58,59,60]. The prognostic value of a single SUMO machinery component can also vary between cancer (sub)types, and, e.g., the value for PC2 [61,62] and PIAS4 [63,64] as prognostic biomarkers in breast cancer and for PIAS3 [65,66] in mesothelioma remains ambiguous (Table 1). Bioinformatic analyses of the SUMO machinery protein and mRNA levels, and their association with prognosis, utilizing datasets from various databases are generally well in line with the other published analyses of often smaller sample sizes [67,68,69] (and Supplementary Table S1). Mass spectrometry-based methods might decrease variability in protein level analyses caused by the use of different antibodies, whereas IHC allows analysis of the subcellular localization of a protein, which might be crucial for the prognostic significance of the SUMO machinery components in particular [63,70,71].

### Regulation of SUMO Machinery Expression and Activity in Cancer

Dysregulation of SUMO pathway components in cancer tissues is often detectable already at the mRNA level (Supplementary Table S1). The expression of SUMO machinery components can be epigenetically altered by DNA methylation. For instance, promoter hypomethylation of SENP6 induces expression of SENP6 in hepatocellular carcinoma (HCC) tissues, and elevated SENP6 mRNA and protein levels are associated with promotion of HCC tumorigenesis [72,73]. At the post-transcriptional level, numerous miRNAs are implicated in SUMO regulation, and inverse expression levels of SUMO components and their miRNA-regulators are found in several cancers [74,75,76,77,78]. For example, a low expression of miR-145 is correlated with high expression of SENP1 in prostate cancer cells, and introduction of miR-145 causes cell cycle arrest via inhibition of SENP1 [76]. Oncogenic miRNA-9 and miRNA-181a inhibit PIAS3 in IL-6^high^ breast cancer to promote expansion of early-stage myeloid-derived suppressor cells, resulting in suppression of T-cell immunity [79].

Dysregulation of the expression of SUMO machinery components in protein level can also occur in cancer. Post-translational regulation by, e.g., ubiquitin and subsequent proteasomal degradation may be involved, resulting in alterations in expression levels in cancer [80,81]. Moreover, reactive oxygen species (ROS) typically present in cancer influence protein expression levels of SENPs [82,83]. For instance, the ROS-induced increase in SENP3 level can drive carcinogenesis of head and neck cancer via deSUMOylation and subsequent hyperphosphorylation of STAT3 [83].

In addition to changes in expression levels, catalytic activity of select SUMO pathway components can directly be regulated by conditions typical in cancer, such as hypoxia [84,85]. Hypoxia induces a rapid and reversible inhibition of catalytic activity of SENP1 and SENP3, resulting in altered SUMOylation of a subset of proteins, such as the co-repressor BHLHE40 that is implicated in metabolic reprogramming upon hypoxia.

## 4. Genetic Changes Targeting SUMO Machinery in Cancer

### 4.1. Germline Variants

In breast cancer patients, genetic variation of *UBE2I* can influence the characteristics of tumors [86,87]. Single nucleotide polymorphisms (SNPs) rs7187167, rs11248866, rs8052688, and rs8063 are associated with low-grade (grade 1) tumors, with the strongest tumor grade association displayed by rs7187167 found to be an independent predictor of tumor grade. Moreover, SNP variant rs17354559 of *PIAS3* may be of functional relevance in breast cancer, although the variant was not evaluated experimentally [87]. Furthermore, the genetic variability of SENP1 and SENP2 may play a role in the occurrence of breast cancer [88]. The rs12297820 variant of SENP1 is associated with metastatic status in breast cancer. Women carrying allele C and genotype C/C in the rs12297820 polymorphic site have an increased risk of metastases, while allele T is associated with reduced risk of metastases. Genetic variability of SENP2 may influence the risk and subtype of breast cancer, as allele C and genotype C/C in rs6762208 site correlates with reduced risk, and the A/A genotype is associated with the lack of an estrogen receptor.

Studies performed in a Chinese population indicate that genetic variation of PC2 may influence the occurrence of gastric cancer [89]. Polymorphism rs77447679 is significantly associated with the risk of gastric cancer, as patients carrying the C/A genotype have approximately a 1.7 times higher risk of gastric cancer when compared to the baseline CC genotype, but its mode of action as a potential promoter of tumorigenesis in the context of gastric cancer is still unclear.

Microphthalmia-associated transcription factor (MITF) is a target for a germline missense substitution Mi-E318K at the SUMO consensus motif of MITF (IKQE -> IKQK) required for covalent SUMO binding, leading to the impairment of MITF SUMOylation [90,91]. The prevalence of Mi-E318K mutation is approximately five times higher in patients affected by melanoma, renal cell carcinoma (RCC), or both cancers compared with healthy controls. Overall, the data indicates that mutation of MITF generates a genetic predisposition for the development of melanoma and RCC. MITF plays a critical role in melanocyte development and melanoma carcinogenesis by controlling the expression of several melanoma-associated genes that regulate proliferation and invasion, including *MET5* and *CDKN2A*/*p16INK4A5* [92]. MITF also enhances the transcriptional activity of HIF1α, which is a critical target for kidney cancer susceptibility genes [93,94]. The SUMOylation of MITF represses its transcriptional activity, and accordingly the SUMO-deficient Mi-E318K displays significantly increased overall transcriptional activity when compared to its wild-type counterpart [91]. Interestingly, Mi-E318K and wild-type MITF show similar transcriptional activity on promoters of *MET* and *CDKN2A*, while the activity of HIF1α is promoted more efficiently by Mi-E318K. A specific Mi-E318K signature comprising 32 genes mostly associated with cell growth, proliferation, and inflammation has been identified in RCC cells, implicating that Mi-E318K can alter the transcription of its target genes. Mi-E318K also enhances the invasion, migration, and colony-forming potential of melanoma and RCC cells but does not significantly increase their proliferation rate.

### 4.2. Somatic Mutations

Mutations targeting SUMO machinery have been identified that may contribute to development of cancer. Amplification of distal regions of chromosome 3q is a common event in lung cancer [95]. A network of four genes, SENP2, *DCUN1D1*, *DVL3,* and *UBXN7* was identified as a candidate driver of the 3q26-29 amplicon that occurs in 70–85% of squamous cell carcinomas (SCCs) of the lung [96,97]. In addition to lung cancer, amplifications of 3q26 are also frequent in other epithelial cancers, including head and neck cancer, cervical cancer, and ovarian cancer, which may indicate for SENP2 amplification [97]. As another example, the SENP5-encompassing 3q27.2-q29 region is amplified in 11% of penile squamous cell carcinoma patients, which may indicate enhanced SENP5 activity [98]. Furthermore, the amplification of PIAS1 and/or focal adhesion kinase (FAK) occurs in 8% of non-small-cell lung cancer (NSCLC) cell lines and in a small subset of patient-derived primary NSCLCs and potentially drives lung carcinogenesis [99]. Only a few cases of somatic mutations targeting SUMO pathway components resulting in the expression of altered protein have been characterized. The t(12;15)(q13;q25) chromosomal translocation was found to fuse SENP1 and the mesoderm development candidate 2 (*MESDC2*) in a patient with infantile sacrococcygeal teratoma [100]. The reciprocal fusion genes encode aberrant proteins that display deSUMOylation capacities similar compared with wild-type SENP1. Another example is the fusion between SENP6 (also known as *SUSP1*) and T-cell lymphoma breakpoint associated target 1 (TCBA1) that creates a SENP6–TCBA1 chimeric gene found in a T-cell lymphoblastic lymphoma cell line [101].

Somatic lysine mutations targeting the SUMO acceptor sites of substrate proteins may also lead to the dysregulation of SUMOylation and alter protein function in cancer. Interestingly, lysine mutations have been identified in cancer patients with potential functional relevance, but the extent of lysine mutations leading to aberrant SUMOylation is unknown [102,103].

## 5. SUMOylation Regulates Key Cancer Genes and Hallmark Properties of Cancer Cells

### 5.1. Substrates of SUMO Relevant for Cancer

Oncogenic signaling of growth promoting MYC in cancer cells is dependent on a functional SUMO machinery, indicating that dynamic SUMOylation and deSUMOylation are essential for the regulation of MYC [104]. However, the regulation is context-dependent, and SUMOylation is reported to both upregulate and suppress MYC activity, depending on the specific cell type [105,106,107]. SUMOylation has been suggested to be essential for KRAS/RAF-driven tumorigenesis [108] and is involved in the regulation of major growth-promoting intracellular signaling pathways and their downstream targets. SUMOylation enhances growth-promoting activities of oncogenic AKT, and a mutant form of AKT expressed in several cancers displays elevated levels of SUMOylation [109]. PIAS4-mediated SUMOylation of AMP-activated protein kinase (AMPK) in turn attenuates the inhibitory effect of AMPK towards growth-promoting mTORC1 signaling [110]. SUMOylation of hypoxia inducible factors 1 (HIF1) and 2 (HIF2) is essential for the regulation of several key processes during hypoxia, including angiogenesis, glucose metabolism and erythropoiesis [44,82,85,111,112]. SUMO machinery also mediates NF-κB signaling upon genotoxic stress through the regulation of the NF-κB essential modifier (NEMO) [113]. Furthermore, SUMOylation regulates activation of Wnt/β-catenin signaling via SUMO modification of transducin β-like protein TBL1-TBLR1 complex [114].

SUMOylation modulates several tumor suppressors, such as Rb, as well as p53 and its negative regulator MDM2, regulating cell cycle progression and stress responses [115,116,117,118,119,120]. SUMOylation positively regulates the activity of tumor suppressor PTEN, which is a negative regulator of the oncogenic phosphatidylinositol-3 kinase (PI3K)-AKT pathway and is involved in DNA damage response (DDR) [121]. SUMO machinery influences membrane association and subcellular localization of PTEN, controlling its tumor-suppressive functions in both cytoplasm and nucleus, suppressing tumor growth and, in contrast to other reports, suggesting that SUMOylation is a positive regulator of the PI3K-AKT-mTOR pathway [122,123,124].

### 5.2. SUMOylation in Cellular Processes Relevant for Cancer

The SUMO pathway regulates various essential cellular processes involved in tumorigenesis, including cell cycle progression, stress responses such as DDR, and response to hypoxia, angiogenesis, invasion, stem-like cell properties, and immune responses [44,125,126,127,128,129,130]. For example, PIAS1 and PIAS4 are promoters of double-strand break repair, and SENPs are critical for mediating chromosome structure and homologous recombination as well as nonhomologous end joining [125,131,132,133]. Moreover, SUMOylation influences DNA replication by targeting translesion polymerase eta (polη) to replication forks [134].

SUMO may be involved in the epigenetic regulation of gene expression in cancer. SUMOylation of histones is involved in regulation of chromatin dynamics [135]. SUMOylation may also influence the activity of methyltransferases, such as DNMT1, MLL1/MML2 complex and G9a [136,137,138]. PIAS1 has been shown to control epigenetic silencing of specific gene sets in certain contexts [139,140]. During T-cell differentiation PIAS1 maintains a repressive chromatin state of the *FOXP3* promoter by recruitment of DNA methyltransferases and heterochromatin protein 1, which restricts the differentiation of natural regulatory T-cells (Tregs) [139].

The SUMO pathway regulates angiogenesis in response to hypoxia through modulation of vascular endothelial growth factor (VEGF) expression levels [44,141,142]. Furthermore, SUMOylation is involved in cellular migration and epithelial mesenchymal transition (EMT) via multiple mechanisms [143,144,145,146,147,148]. The SUMO pathway is also implicated in regulation of stem-like cell properties of cancer cells e.g., via PIAS3 and STAT3 [129,149].

SUMO machinery is a critical regulator of innate immune responses via the modulation of type I interferon (IFN) and NF-κB signaling [130,150]. The SUMO pathway regulates the production and activity of IFNs by inhibiting or stimulating the transcription of IFN regulatory transcription factors (IRFs), such as IRF3 and IRF7 [151,152,153,154]. The SUMOylation of IRFs represses the transactivating capacity of IRFs, resulting in diminished transcription of type I IFN genes and attenuation of immune response activation [152,155]. SUMO also impacts IFN production via the modulation of GMP–AMP synthase (cGAS) and the stimulator of interferon genes (STING) [156,157]. SUMO also influences the activity of NF-κB signaling regulators, NEMO and IκBα, affecting immune response activation [158,159,160,161,162]. SENP3-mediated deSUMOylation regulates antitumor immune response functions of Tregs, macrophages, and dendritic cells [163,164,165]. The loss of SENP3 deSUMOylase activity in Tregs results in dysregulation T-cell homeostasis and SENP3 deficiency in macrophages facilitates macrophage polarization towards the pro-tumor M2 subtype. Overall, SUMOylation is considered to have a net inhibitory effect on immune response activation [150,166,167]. Depletion of Ubc9 or SAE1/2 induces a strong inflammatory response and enhances protection of viral infections in hematological xenograft mouse models.

## 6. SUMOylation in Hematologic Malignancies and Solid Tumors

The SUMO pathway contributes to the tumorigenesis of various cancer types in a context-dependent manner. Here, we discuss the best-characterized examples of the role of the SUMO pathway in the tumorigenesis of hematological malignancies and solid tumors.

### 6.1. Acute Promyelocytic Leukemia

**Figure 2:**
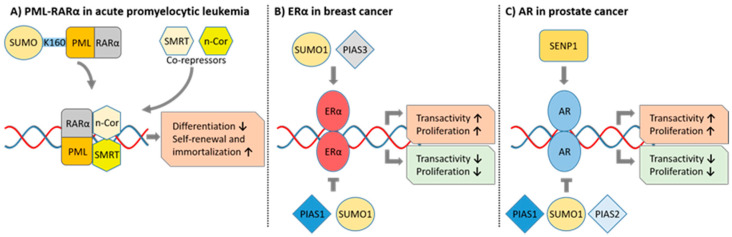
Examples of SUMO-modification regulation of key oncogenic substrates. (**A**) SUMOylation of PML-RARα in K160 and recruitment of co-repressors is required for APL cell differentiation blockade, self-renewal and immortalization. (**B**) PIAS3 promotes transactivation of estrogen receptor α (ERα) and proliferation of breast cancer cells via SUMO-dependent and independent mechanisms, whereas PIAS1 has a predominantly repressive effect on ERα. (**C**) SENP1-mediated deSUMOylation of androgen receptor (AR) promotes AR-dependent transactivation and proliferation of prostate cancer cells that is counteracted by PIAS1- and PIAS2-induced SUMO1-modification of AR.

Acute promyelocytic leukemia (APL) was one of the first clinical links between SUMO modification and cellular transformation (Figure 2A). APL is a subtype of acute myeloid leukemia (AML) characterized by the reciprocal chromosome translocation t(15;17) that creates the oncogenic fusion protein promyelocytic leukemia (PML)-retinoic acid receptor α (RARα) [168,169,170,171]. The expression of PML-RARα disrupts the integrity of PML nuclear bodies, which are known hotspots for SUMOylation [172,173,174]. RARα is a hormone-regulated nuclear receptor that regulates transcription of differentiation-associated genes by recruiting corepressors, such as SMRT and N-CoR, to repress cellular differentiation [171]. RARα-induced gene repression is counteracted upon the binding of its natural ligands, such as all-trans-retinoic acid (ATRA) that alleviate RARα-maintained differentiation repression [171,175]. The oncogenic fusion protein PML-RARα binds corepressors with a higher affinity than its wild-type counterpart and physiological levels of ATRA in APL patients are insufficient to release PML-RARα-maintained repression of differentiation genes, resulting in a myeloid differentiation blockade [176]. The APL differentiation block was initially attributed only to the formation of PML-RARα homodimers, and the subsequent enhancement of corepressor binding and repression of target genes [177]. However, later studies revealed that the SUMOylation of PML-RARα plays a major role in the pathogenesis of APL. The SUMOylation of PML at the SUMO site K160 is essential for APL initiation and maintenance, and the inactivation of K160 impairs the differentiation blockade and immortalization of APL cells [178] (Figure 2A). Mouse primary hematopoietic progenitor cells transduced with SUMO-deficient (K160R) PML-RARα grow only for a limited period, and its expression in mice induces myeloid hyperplasia that does not progress to APL, indicating that APL cell immortalization and differentiation block is SUMO-dependent.

Differentiation therapy with ATRA in combination with arsenic trioxide (ATO) has emerged as an effective therapeutic strategy for treatment of APL [171]. ATRA and ATO synergize to induce differentiation and death of APL cells, in which ATRA alleviates the repressive chromatin environment and induces degradation of the PML-RARα [179,180,181]. ATO enhances the binding of Ubc9 to both PML-RARα and wild-type PML, triggering their SUMOylation and recruitment of ubiquitin E3 ligase ring finger protein 4 (RNF4) [182]. Mechanistically, ATO triggers SENP1-dependent exchange of SUMO1 to SUMO2 at Lys65 of PML, enhancing the formation of PML nuclear bodies and the conjugation of SUMO2 to Lys160, which results in RNF4-mediated polyubiquination and proteasomal degradation of PML-RARα in nuclear bodies [182,183]. Overall, the data indicate that SUMOylation plays major roles in both APL development and its remedy.

### 6.2. Acute Myeloid Leukemias Other Than APL

While ATRA and ATO-based differentiation therapies are an effective treatment strategy for APL, they have failed to show significant efficacy in clinical trials of non-APL AML patients [184]. This has been attributed to differences in epigenetic and transcriptional profiles of AML subtypes. SUMOylation has been shown to repress the expression of various ATRA-responsive genes, indicating that the SUMO pathway restricts ATRA’s ability to induce expression of genes associated with myeloid differentiation in non-APL AMLs [185]. Combining ATRA treatment with pharmacological inhibitors of SUMOylation, 2-D08 (inhibitor of Ubc9), or anacardic acid (inhibitor of SAE1/2), results in enhanced expression of various ATRA-responsive genes associated with myeloid differentiation, including *RARA*, *CEBPA,* and *TNSFS10* [185]. A similar increase in expression of ATRA-responsive pro-differentiation genes is observed when SENP2 or SENP5 is overexpressed in AML cells [185]. ATRA synergizes with SUMOylation inhibitors to induce morphological changes of myeloid differentiation, including nuclear lobulation and appearance of cytosolic granules in several non-APL AML cell lines and primary AML cells. A combination of 2-D08 and ATRA inhibits the proliferation of AML cell lines, reduces proliferation of primary AML cells, and restricts tumor growth in immunodeficient mice subcutaneously xenografted with U937 cells. Moreover, 2-D08 induces mitochondrial-mediated apoptosis through an accumulation of ROS that is likely triggered by deSUMOylation of NOX2 [186]. Taken together, the inhibition of SUMOylation in combination with ATRA or other differentiation agents may be an attractive novel therapeutic strategy for treatment of non-APL AMLs.

Additionally, the SUMO pathway is critically involved in the regulation of DDR of AML cells via nucleophosmin (NPM) and human coilin-interacting nuclear ATPase protein (hCINAP) [187]. NPM is the most frequently mutated gene in non-APL AMLs [188,189]. Generation of double-strand breaks triggers the SUMOylation of NPM, which is required for its interaction with BRCA1 and accumulation of BRCA1 foci to damage sites in the early stage of DDR [187]. During the later stages of DDR, hCINAP is recruited to damage sites where it binds with SENP3, which enhances SENP3-mediated deSUMOylation of NPM and decreases the recruitment of repair proteins to prevent excessive repair [187,190]. The lower expression of hCINAP is associated with better prognosis in AML patients [187]. Depletion of hCINAP in patient-derived xenograft AML mice results in higher sensitivity to chemotherapy, elevated levels of DNA damage and cell death, and mitigation of AML progression. Thus, hCINAP expression levels affect DDR via the regulation of NPM SUMOylation, making hCINAP a potential therapeutic target in AML patients.

### 6.3. Lymphomas

The SUMO pathway influences development of both B- and T-cell lymphomas [106,191,192]. The aberrant activation of the SUMOylation pathway is a hallmark of MYC-driven B-cell lymphomas [106,191]. MYC expression induces the transactivation of several SUMO machinery components, including Ubc9 and PIAS1 to promote global hyper-SUMOylation in P493-6 B lymphoma cells and several Burkitt’s lymphoma cell lines [191]. Furthermore, PIAS1-induced SUMOylation stabilizes MYC, and PIAS1 is implicated in facilitating transactivation-associated phosphorylation of MYC in B-cell lymphomas [106]. The silencing of SAE1, SAE2 or PIAS1 and pharmacological inhibition of SUMOylation with anacardic acid or 2-D08 reduces proliferation and induces apoptosis in various MYC-driven B lymphoma cell lines, and the silencing of SAE2 or PIAS1 significantly impairs B-cell lymphomagenesis in mouse xenograft models [106,191]. Moreover, SUMOylation contributes to pathogenesis of T-cell lymphomas expressing the oncogenic NPM-anaplastic lymphoma kinase (NPM-ALK) fusion protein via SUMO-dependent post-translational stabilization of NPM-ALK [192]. SUMO1–3 levels are upregulated in NPM-ALK+ T-cell lymphoma cell lines and ALK+ T-cell lymphoma patient tumors, whereas SENP1 levels are significantly downregulated. The introduction of SENP1 expression induces deSUMOylation of NPM-ALK, and the subsequent ubiquitination and proteasomal degradation of NPM-ALK, decreasing the viability, proliferation and anchorage-independent colony formation in NPM-ALK+ T-cell lymphoma cell lines. Thus, the inhibition of SUMOylation should be explored as a therapeutic option for treatment of MYC-dependent B-cell lymphomas and NPM-ALK+ T-cell lymphomas.

### 6.4. Multiple Myeloma

Multiple myeloma (MM) is a hematological malignancy characterized by the clonal expansion of plasma cells that produce monoclonal immunoglobulin (Ig) and Ig fragments, termed M-protein or paraprotein, which are detectable in the serum or urine [193]. The SUMO pathway is often dysregulated in MM patients, and high SUMOylation is associated with adverse patient outcome [194]. The overexpression of Ubc9, SENP1, PIAS1, and SUMO-inducer p14ARF are frequently observed in MM patient samples and MM cell lines [194,195]. While p14ARF is not traditionally considered as a part of the classical SUMO machinery, it interacts with Ubc9 to trigger the SUMOylation of many of its binding partners, such as NPM and MDM2 [118,196,197]. The induction of Ubc9 expression in MM is associated with increased proliferation and apoptotic resistance, and enhanced adhesion of MM cells to bone marrow stroma cells (BMSCs) [194]. The adhesion of MM cells to BMSCs induces interleukin-6 (IL-6) secretion and activation of the NF-κB pathway, which further stimulates proliferation of MM cells [194,198,199,200]. MM cells transfected with a functionally inactive dominant-negative form of Ubc9 display a decreased proliferation rate, are more sensitive to radiation-induced apoptosis, and have a weaker proliferative response in the presence of BMSCs when compared to wild-type Ubc9-expressing MM cells [194]. Aberrant IL-6 signaling upregulates expression of SENP1 at least partly through the induction of the JAK-STAT-signaling pathway, and the treatment of MM cells with JAK-STAT inhibitor AG490 diminishes IL-6-mediated upregulation of SENP1 [195]. The knockdown of SENP1 with shRNA results in significantly increased SUMO2 levels and blocks the IL-6-induced activation of NF-κB family member p65, implicating that SENP1 positively regulates NF-κB signaling via SUMO modification of NF-κB family members. Furthermore, the knockdown of SENP1 reduces viability, proliferation, and colony-forming potential, as well as induces apoptosis in MM cells.

The downregulation of SENP2 in MM patients is associated with drug resistance against bortezomib, which is a widely used proteasome inhibitor for treatment of MM [56]. In MM patients, low SENP2 expression levels result in aberrant activation of NF-κB signaling [56]. The NF-κB pathway is normally regulated by a negative feedback mechanism, where NF-κB activates expression of SENP2, which in turn inhibits NF-κB signaling. However, the silencing of SENP2 in MM through yet-unknown mechanisms results in enhanced SUMOylation of IκBα, activation of NF-κB signaling and development of bortezomib resistance in MM patients. Identifying SUMO ligases that regulate IκBα is important, as they may serve as novel therapeutic targets in bortezomib-resistant MM. Overall, the dysregulation of SUMO pathway is widespread in MM, which encourages further studies to characterize which SUMO components or their regulatory targets have most therapeutic or prognostic value. Pharmacological inhibition of SUMOylation and SENP1 may have therapeutic potential for treatment of MM.

### 6.5. Breast Cancer

The SUMO pathway has a multifunctional role in the regulation of growth and the invasion and metastasis of breast cancer cells [201,202,203]. In hormone receptor (HR)-positive breast cancer, the SUMO pathway is an essential regulator of estrogen receptor α (ERα) (Figure 2B) [204]. PIAS1 and PIAS3 modulate transactivation of ERα through its direct SUMOylation that occurs strictly in the presence of the hormone, and via the SUMOylation of ERα cofactors (Figure 2B). However, PIAS1, PIAS3, and Ubc9 also regulate ERα transactivation in a SUMO-independent manner. The induction of SUMO-deficient ERα to COS-7 cells reduces the transactivation of ERα, indicating that SUMOylation of ERα may promote its transactivation. However, excessive SUMOylation of ERα is linked to repression of ERα transactivation. Tumor suppressor MEL-18 regulates ERα activity by suppressing the SUMOylation of ERα transactivators p53 and Sp1 to promote ERα and progesterone receptor (PR) activity, and MEL-18 may be a prognostic marker and predictor of response to antihormonal therapy in breast cancer [205]. SUMOylation is also important for mechanisms of action of antiestrogens in the treatment of HR-positive breast cancer [206]. Interestingly, the upregulation of PIAS1 is associated with epigenetic silencing of breast cancer-associated genes, including *ESR1* (ERα) [140]. Thus, PIAS1 can both upregulate and downregulate ERα activity in breast cancer. PIAS1 also promotes the SUMOylation of transcriptional regulator SnoN that suppresses TGFβ-induced EMT in ER-positive MCF7 cell-derived organoids [148]. In contrast, PIAS3 stimulates proliferation of ER-positive breast cancer cells, and PIAS3 acts as a co-activator to regulate NR2E3-mediated activation of *ESR1* (ERα) expression [207,208]. PIAS3 is involved in the regulation of receptor tyrosine kinase ErbB4 [209,210]. ErbB4 can be proteolytically cleaved to release an intracellular domain (ICD) that acts as a transcriptional co-regulator implicated in the regulation of differentiation and proliferation of mammary epithelial cells [211,212,213]. PIAS3 interacts with ErbB4 ICD to induce its SUMOylation, which promotes its nuclear accumulation and may facilitate autokinase activity of ErbB4 [209,210]. Interestingly, nuclear ErbB4 immunoreactivity is associated with a poor survival of ER-positive patients when compared to cell surface expression [213]. Thus, inhibition of PIAS3 may be an attractive therapeutic target in HR-positive breast cancer, but although the targeting of PIAS1 in breast cancers overexpressing PIAS1 could potentially relieve PIAS1-mediated epigenetic repression, the role of PIAS1 as a repressor of invasion must be considered.

In triple-negative breast cancer (TNBC), PIAS1 promotes the SUMOylation of SnoN that suppresses TGFβ-induced EMT in TNBC MDA-MB-231 cell-derived organoids similarly to HR-positive breast cancer models [148,214]. Moreover, the induced expression of PIAS1 suppresses TGFβ-induced activation of invasion promoter matrix metalloproteinase 2 (MMP2) in MDA-MB-231 cells, and PIAS1 knockdown in TGFβ-treated MDA-MB-231 cell organoids results in morphological changes associated with invasion [214]. However, PIAS1 is essential for viability of MYC-dependent breast cancer cells, as the silencing of PIAS1 reduces the proliferation of MYC-dependent MDA-MB-231 cells, but not MYC-independent MCF7 cells [106]. MYC signaling is elevated in TNBC, and the interaction of PIAS1 with MYC drives its SUMOylation-dependent stabilization and upregulation of MYC target gene transcription [104,106]. PIAS3 inhibits proliferation and EMT in MDA-MB-231 TNBC cells in contrast to pro-proliferation effects observed in ER-positive breast cancer [203,208]. PIAS3 enhances the SUMOylation of E3 ubiquitin ligase Smurf2, facilitating the degradation of TGFβ receptors, and subsequently suppressing the invasive growth of MDA-MB-231 cells [203]. The knockdown of PIAS3 induces invasion-associated morphological changes in MDA-MB-231 organoids, indicating that Smurf2′s ability to limit EMT is SUMO-dependent. Overall, the contradictory observations of PIAS3 effects in TNBC and HR-positive breast cancer may suggest that different effects of PIAS3 on breast cancer cells may involve ER, whereas therapeutic targeting of SUMOylation and PIAS1 may be an effective strategy for the treatment of MYC-dependent breast cancers. In addition, the overexpression of PML is associated with high invasive tumor grade in TNBC patients, suggesting PML as a putative therapeutic target in TNBC [215].

SENP1 and SENP5 are implicated in regulation of invasiveness of TNBC [202,216]. SENP1 expression is upregulated in TNBC tissues compared to normal breast tissues and non-TNBC tissues [216]. Silencing of SENP1 reduces proliferation, invasion and colony formation of TNBC cell lines and reduces tumor size and lung metastases in a TNBC mouse xenograft model. High expression of the SENP5 gene is associated with poor prognosis in breast cancer patients [202]. The silencing of SENP5 has a profound inhibitory effect on the invasiveness of TNBC MDA-MB-231 and MDA-MB-436 cells, which is not observed in less invasive ER-positive MCF7 and T47D cells. The knockdown of SENP5 in TNBC cells significantly reduces the level of MMP9 and decreases levels of total, phosphorylated, and SUMOylated TGFβRI, which is a stimulator of invasion at later stages of breast cancer. However, the exact mechanism of how SENP5 regulates TGFβRI, and invasion remain to be elucidated. Pharmacological inhibition of SENP1 and SENP5 may have therapeutic potential for treatment of TNBCs overexpressing SENP1 or SENP5.

Lastly, expression of long splice variant of SENP7 (SENP7L) is associated with the promotion of EMT in both ER-positive breast cancer and TNBC and its expression is upregulated in breast cancer tissues compared to normal breast epithelia tissues [201]. SENP7L-induced deSUMOylation of epigenetic remodeler heterochromatin protein 1α (HP1α) promotes EMT by maintaining HP1α at a hypo-SUMOylated state, relieving its repression of mesenchymal promoting genes.

### 6.6. Prostate Cancer

PIAS family members promote prostate cancer (PCa) tumorigenesis through the inhibition of CDK p21 levels and modulation of androgen receptor (AR) signaling, which is the most important known growth promoting pathway in PCa (Figure 2C) [217,218,219]. PIAS1, PIAS2, PIAS3, and PIAS4 are co-regulators that selectively enhance or repress transcription of AR target genes via both SUMO-dependent and -independent mechanisms [217,218,220,221,222,223]. For instance, PIAS1- and PIAS2-induced SUMOylation of AR represses its transcriptional activity. However, PIAS1 can also enhance AR-mediated upregulation of growth-promoting genes independent of its SUMO-ligase activity to drive proliferation PCa cells, and high PIAS1 expression is associated with poor survival. In contrast, PIAS3 represses the transcriptional activity of STAT5 that is a regulator of growth and viability of PCa cells, and is constitutively active in PCa and associated with high histological grade [224,225]. PIAS3-mediated SUMOylation of STAT5 is reported to inhibit its activation-associated phosphorylation in COS-1 cells, which may indicate that PIAS3 mode of action on STAT5 repression is partially SUMO-dependent [226]. Interestingly, a subset of PCa cells express an N-terminally cleaved form of STAT5 that cannot be targeted by PIAS3 [227]. However, the extend of SUMOylation-dependent effects in PIAS-mediated outputs on PCa is not clear.

SUMO machinery can both repress and activate AR signaling in a target gene-selective manner [228,229,230]. SUMO1-modification inhibits AR transactivation capacity, whereas SUMO2 and SUMO3 are reported to stimulate AR activity in PCa cells, although likely independent of direct AR SUMOylation [228,229]. Overall, the SUMOylation of AR is shown to repress transcription of various AR target genes, while SENP1-induced deSUMOylation is linked to the promotion of AR transactivation capacity [230] (Figure 2C). The expression of SENP1 is frequently observed in PCa and precancerous prostatic intraepithelial neoplasia (PIN) tissues, and the overexpression of SENP1 correlates with increased aggressiveness and recurrence of PCa [142,231]. Elevation of SENP1 mRNA is selectively mediated through the activation of AR in PCa cells [232]. SENP1 also enhances AR-dependent transcription through the deSUMOylation of histone deacetylase 1 (HDAC1), diminishing its ability to repress AR [233]. HDAC4 is another SUMO-dependent repressor of AR and may be regulated by SENPs [234].

In addition to the regulation of AR transactivation capacity, SENP1 stimulates proliferation of PCa cells via an AR-independent induction of cyclin D expression, and the silencing of SENP1 results in the downregulation of cyclin D and inhibition of cell growth [231]. During hypoxia, SENP1 deSUMOylates and stabilizes HIF1α, promoting angiogenesis and invasion via the upregulated expression of MMP2, MMP9, and VEGF [44,141,142]. SENP1 expression in PCa patient samples positively correlates with the expression of MMP2 and MMP9 that are frequently overexpressed in prostate cancer [142]. SENP1 promotes invasion also through the deSUMOylation of reptin, diminishing its repressive effect on invasion and metastasis [235]. Furthermore, the SUMOylation status of reptin modulates the invasive activity of cancer cells with metastatic potential.

The genetic silencing of SENP1 or pharmacological inhibition with momordin Ic or triplotide reduces colony formation, migration, and invasion in PCa cell lines, as well as suppresses tumor growth in mouse PCa xenograft models [142,236,237]. Interestingly, SENP1 positively regulates the stability of tumor suppressor PTEN by blocking its SUMO-dependent ubiquitylation and degradation [238]. PTEN is considered to serve as a barrier for SENP1-induced PCa tumorigenesis, and the inhibition of SENP1 may be a therapeutic option for treatment of aggressive PTEN-deficient PCa. SUMOylation controls the subcellular localization of hexokinase 2 (HK2), which is a regulator of glycolysis and has oncogenic potential when it binds to mitochondria [239]. SENP1-induced deSUMOylation stimulates HK2 binding with mitochondria to promote metabolic reprogramming, supporting proliferation of PCa cells and protecting them from chemotherapy-mediated apoptosis. The knockdown of Ubc9 increases the binding of HK2 to mitochondria, whereas the knockdown of SENP1 reduces binding to mitochondria, indicating that SUMOylation is necessary for protection against PCa tumorigenesis. High expression of both SENP1 and HK2 is associated with poor prognosis and poor response to docetaxel-based chemotherapy in PCa patients. Moreover, SENP1 regulates deSUMOylation of spliceosome factor USP39 that is implicated in PCa tumorigenesis [240]. The introduction of SUMO-deficient USP39 enhances its ability to promote proliferation of both androgen-dependent and -independent PCa cells, suggesting that deSUMOylation can enhance its oncogenic activity. Taken together, pharmacologic inhibition of SENP1 may be an attractive option for the treatment of PCa due to SENP1′s various oncogenic functions.

Lastly, androgen-dependent GTPase-activating protein-binding protein 2 (G3BP2) interaction with RanBP2 mediates SUMOylation of p53 that induces its export to cytoplasm, repressing tumor-suppressive functions of p53 [241]. Elevated cytoplasmic p53 localization correlates with increased G3BP2 expression and is associated with disease progression to the hormone-refractory state and poor prognosis. The silencing of RanBP2 inhibits dihydrotestosterone (DHT) mediated nuclear export of p53, and the depletion of G3BP2 increases nuclear levels of p53 and reduces tumor growth in PCa mouse xenograft models. Thus, therapeutic targeting of RanBP2 or G3BP2 may offer therapeutic advantages in PCa expressing high levels of cytoplasmic p53.

### 6.7. Kidney Cancer

Renal cell carcinomas (RCCs) are characterized by diminished activation of E3 ubiquitin ligase VHL [94]. Prolyl 4-hydroxylases hydroxylate HIFs and facilitate the binding of E3 ubiquitin ligase VHL to HIFs that promotes their ubiquitination and proteasomal degradation in normoxia, but in hypoxic conditions HIFs are stabilized [242,243]. HIFs regulate the expression of several genes involved in energy metabolism, angiogenesis, and cell proliferation. In RCC, the stabilization of HIFs due to lack of VHL activity supports tumor growth, and the HIF-VEGF axis is a target for therapeutics used in the treatment of RCC patients [94]. SUMOylation-related deregulation of HIF1α and HIF2α transactivation are strongly implicated in development of RCCs [44,244,245]. Regulatory axis of VHL, SENP1, and SUMO-enhancer RSUME is essential for the modulation of HIF activity in response to hypoxia. Hypoxia induces SENP1-mediated deSUMOylation of HIFs, promoting the stabilization and transactivation of both HIF1α and HIF2α by protecting them from VHL-mediated proteasomal degradation [44,244]. Tissues from clear-cell RCC (ccRCC) patients expressing high levels of SENP1 display increased expression of various glycolytic enzymes, and high SENP1 expression is associated with poor prognosis in ccRCC patients [245]. In addition, protein levels of HIF1α and HIF2α are upregulated in ccRCC tumors when compared to normal tissues. The knockdown of SENP1 decreases proliferation of RCC4/VHL cells in hypoxic conditions and reduces mRNA levels of key glycolytic enzymes such as *PGK1*, *PFK1,* and *ALDOA*, and hypoxia-response genes VEGF and *MMP-9* more potently under hypoxia compared to normoxia. Mechanistically, SENP1 enhances aerobic glycolysis through deSUMOylation and stabilization of HIF1α, which in turn upregulates transcription of glycolysis-related genes in ccRCC. Transfection of RCC4/VHL cells with SUMO-defective HIF1α results in increased mRNA levels of HIF1α transactivation target genes, supporting that deSUMOylation of HIF1α drives the upregulation of glycolytic enzymes and other downstream genes. Thus, increased SENP1 and HIF1α activity promote RCC development at least in part by inducing a metabolic shift towards aerobic glycolysis.

RSUME is reported to stabilize and enhance transactivation of HIF1α via induction of HIF1α SUMOylation in response to hypoxia, which partially contradicts the model in which HIF1α is activated by deSUMOylation [111,112]. However, later studies advocated for an alternative mechanism for RSUME-mediated activation of HIF1α and HIF2α, in which RSUME counteracts the negative regulator of HIF, E3 ubiquitin ligase VHL [246,247]. RSUME interacts with VHL, which promotes the SUMOylation of VHL and inhibits its assembly, thus blocking the proteasomal degradation of HIF1/2α [246]. RSUME is also suggested to potentiate VHL mutant phenotype [247]. Taken together, the targeting of the SUMO pathway and SENP1/HIF1α axis with pharmacological inhibitors of SENP1 may have therapeutic potential in treatment of RCC.

### 6.8. Lung Cancer

PIAS1-mediated SUMOylation induces autophosphorylation and activation of FAK, which is a regulator of cytoskeleton remodeling, mitogenic signaling, and cell survival [248]. PIAS1 also regulates the subcellular localization of FAK [99]. Co-amplification PIAS1 and FAK is reported in a subset of non-small-cell lung cancers (NSCLCs). PIAS1-mediated nuclear recruitment of FAK promotes DNA damage repair, which may provide a survival advantage to genomically unstable NSCLC tumors. Hypoxia-induced Ubc9- and PIAS4-mediated SUMOylation of migration regulator Slug enhances its transcriptional repression activity by recruitment of corepressors, decreasing expression of Slug downstream targets, such as E-cadherin [249]. SUMOylation of Slug promotes migration, invasion, and metastasis in lung cancer cells. The overexpression of Ubc9 or Slug is associated with poor prognosis in NSCLC patients, whereas a combined low expression level of Slug and Ubc9 is linked to better survival. PIAS3 in turn suppresses the proliferation of lung cancer cells through SUMO-independent inhibition of STAT3 and PI3K/AKT signaling, as well as via the promotion of apoptosis [250,251,252,253]. PIAS3 protein levels are often downregulated, and low PIAS3 expression is associated with poor survival of squamous cell lung cancer patients [80,254]. Induced overexpression of PIAS3 in NSCLC cell lines decreases proliferation through suppression of AKT phosphorylation and reduced transactivation of STAT3 [251,252]. PIAS3 overexpression promotes STAT3- and p53-independent apoptosis through activation of intrinsic apoptotic pathway in NSCLC cell lines [253]. PIAS3 overexpression also potentiates anti-proliferative effects of erlotinib, which is an EGFR inhibitor used in NSCLC therapies [252]. Moreover, SENP2 is frequently overexpressed in lung cancer due to the prevalence of the 3q26-29 amplicon [96,97]. The network of SENP2, *DCUN1D1*, *DVL2,* and *UBXN7* regulates proliferation of lung cancer cells that carry the 3q26-29 amplicon, as knockdown of any of the four proteins leads to inhibition of growth [96]. High expression of the three-gene signature of SENP2, *DCUN1D1,* and *DVL3* may also predict benefit from adjuvant chemotherapy in SCC patients. Thus, amplified PIAS1 and SENP2 are potential therapeutic targets in lung cancer, whereas the expression level of PIAS3 may predict sensitivity towards select treatments.

### 6.9. Hepatocellular Carcinoma

SENP1 regulates hepatocellular carcinoma (HCC) carcinogenesis through deSUMOylation of ubiquitin-conjugating enzyme E2T (UBE2T) and HIF1α [255,256]. SENP1-mediated deSUMOylation of UBE2T promotes HCC development through activation of the PI3K-AKT pathway, and both SENP1 and UBE2T are overexpressed in most HCC tissues and hepatoma cell lines. A positive feedback loop between SENP1 and HIF1α is implicated to contribute to the maintenance of HCC cell stemness under hypoxia. The knockdown of SENP1 decreases proliferation, colony formation, invasion, and stemness of HCC cells, and reduces tumor growth in mouse xenograft models, indicating SENP1 as a potential therapeutic target in HCC. PC2-mediated SUMOylation and transactivation of HIF1α drives angiogenesis and HCC tumorigenesis via hypoxia-induced upregulation of VEGF expression [68]. Moreover, SUMOylation of Shp2 promotes ERK-mediated promotion of HCC cell and tumor growth [257]. Taken together, the inhibition of SUMOylation and SENP1 may have therapeutic potential in HCC.

### 6.10. Gliomas

Protein levels of SUMO1, SUMO2/3 and Ubc9 are upregulated in patient samples derived from astrocytic tumors, with a moderate upregulation in low-grade astrocytoma (grade 2) and anaplastic astrocytoma (grade 3), and a massive upregulation in glioblastoma multiforme (GBM; grade 4) [258]. Furthermore, high protein expression of SAE1 is associated with poor prognosis of glioma patients [67]. Silencing of SUMO1–3 reduces proliferation and clonogenic survival, and suppresses DNA repair and synthesis in GBM cells [258]. Mechanistically, SUMO1-modification of CDK6 blocks its ubiquitin-mediated proteosomal degradation to drive proliferation of GBM cells [259]. Moreover, SAE1 enhances SUMO1-modification and activation-associated phosphorylation of AKT, promoting the proliferation and migration of glioma cells [67]. Interestingly, SAE inhibitors ginkgolic and anacardic acid fail to decrease global SUMOylation in GBM cell lines, which may be due to especially high levels of SUMOylation in GBM [260]. Topotecan, an inhibitor of DNA topoisomerase I, can significantly reduce global SUMOylation in GBM cell lines [260]. Topotecan suppresses cell cycle progression via CDK6 and alters HIF1α-associated metabolic programming by the inhibition of pentose phosphate pathway and glycolytic metabolism in GBM.

PIAS3 is implicated in the regulation of stem-like cell properties of GBM cells [129,149]. SMAD6 induces ubiquitin-mediated degradation of PIAS3 in GBM cells to enhance STAT3-mediated proliferation and stem-like cell initiation, whereas TRIM8 represses PIAS3 through a similar mechanism to maintain STAT3-mediated stemness and self-renewal of GBM stem-like cells. PIAS3 is downregulated in GBM tissues, likely due to ubiquitin-mediated proteosomal degradation [81]. Intriguingly, SUMO1, but not SUMO2/3, appears to play a major role for maintenance of GBM stem-like cells, and PML is the major substrate for SUMO1 in these cells, facilitating stabilization of c-Myc [261]. Overall, the inhibition of SUMOylation may be an attractive strategy for treatment of GBM.

## 7. Therapeutic Targeting of the SUMO Pathway

The SUMO pathway is increasingly viewed as a potential therapeutic target in cancer [262], and several inhibitors of SUMO E1-activating, E2-conjugating and deconjugating enzymes have been identified (Table 2).

### 7.1. SUMO E1 Inhibitors

Ginkgolic acid and its structural analog, anacardic acid, were the first compounds identified to inhibit the SUMO pathway [263]. Ginkgolic acid inhibits SUMOylation by binding to SAE and blocks the formation of E1-SUMO intermediates. Other natural products similarly targeting SUMO E1 activity include antibiotic kerriamycin B, ellagitannin davidiin and tannic acid (Table 2) [264,265,266]. However, natural source-derived SAE inhibitors function mainly in micromolar range and can have several non-SUMO-related effects in cells, which may complicate the interpretation of their anti-cancer mechanisms. Recent advances in the development of synthetic inhibitors have enabled more specific and efficient targeting of the SUMO pathway. Synthetic inhibitors of SAE include compound 9, compound 21, various pyrazole and thiazole urea-containing compounds, ML-792, COH000, ML-93, several ginkgolic acid derivatives, and most recently, TAK-981 [267,268,269,270,271,272,273,274,275]. For example, compound 21 and COH000 act in a similar way as the natural SAE inhibitors. In contrast, ML-93, ML-792, and its derivative TAK-981 inhibit SAE activity by forming a covalent adduct with SUMO, which is catalyzed in an ATP-dependent manner by SAE1/2 itself. TAK-981 is an especially potent inhibitor of SUMOylation that can activate a strong antitumor immune response [275,276,277,278]. TAK-981 reduces proliferation of HCT116 colon cancer cells in vitro and significantly decreases tumor volumes in HCT-116 and OCI-Ly10 mouse xenograft models in vivo. TAK-981 treatment triggers the upregulation of type I IFN signaling in immune cells, as well as IFN-dependent activation of macrophages, T-cells, natural killer (NK) cells, and dendritic cells.

### 7.2. SUMO E2 Inhibitors

Pharmacological targeting of Ubc9 has generated much interest in cancer research, as it is the sole E2-conjugating enzyme known in mammals. Several inhibitors of Ubc9 have been identified, including natural product spectomycin B1 and its structural relatives chaetochromin A and viomellein, semisynthetic SUMO-based Ubc9 inhibitors (SUBINs), as well as synthetic inhibitors GSK145A, compound 2 and 2-D08 (Table 2) [279,280,281,282,283]. Antibiotic spectomycin B1 binds to Ubc9 blocking the formation of the E2-SUMO intermediates [279]. Spectomycin B1 treatment suppresses β-estradiol-dependent proliferation of MCF7 breast cancer cells. SUBINs are high-affinity SUMO2 variants that bind to Ubc9 to inhibit poly-SUMO chain formation [283]. GSK145A acts as a SUMO substrate mimic that is strongly targeted for SUMOylation, resulting in the depletion of available SUMO-Ubc9, whereas compound 2 directly binds to Ubc9 [280,282]. 2-D08 inhibits the transfer of SUMO proteins from Ubc9 to target substrates [281]. 2-D08 displays antiproliferative activity in several AML cell lines and bladder cancer T24 and 5637 cell lines, as well as suppresses migration of K-Ras-mutated MiaPaCa-2 pancreatic cancer cells [185,284,285].

### 7.3. SENP Inhibitors

SUMO-deconjugation machinery is frequently dysregulated in cancer, and high expression of different SENP proteases is associated with tumorigenesis of several cancers (Table 1 and Table S1), indicating SENPs as potential therapeutic targets [286]. Triplotide and momordin Ic (Mc) are natural SENP1 inhibitors that have shown anticancer activity towards prostate cancer (Table 2) [236,237]. Triplotide and momordin Ic decrease the proliferation of LNCaP and PC3 cell lines and inhibit tumor growth in PC3 xenograft mice models. In addition, various synthetic inhibitors of SENP1 have been developed, including compound 38, compound J5, compound 4 (GN6958), compound 13m, and compounds 6, 7, and 10 [287,288,289,290,291]. However, in vivo data of synthetic SENP1 inhibitors in cancer models are lacking. To date, only a few specific SENP2 inhibitors have been discovered, including compounds 69, 117, and ebselen [292,293]. URB597, a fatty acid amide hydrolase inhibitor, indirectly inhibits SENP3 by decreasing levels of ROS in rats with chronic cerebral hypoperfusion [294]. JCP666, VEA260, VEA499, compound 1, compound 3, and antibiotic streptonigrin inhibit activities of both SENP1 and SENP2 simultaneously, whereas SI2 additionally inhibits SENP3 [240,295,296,297,298,299]. Various benzothiophene-2-carboxamide derivatives simultaneously inhibit activities of SENP1, SENP2, and SENP5 [300]. Lastly, SPI-01 targets SENP1, SENP2, and SENP7, while VEA561 inhibits SENP2, SENP6, and SENP7 [295,301].

### 7.4. Current Stage of Clinical Development of SUMO Pathway Inhibitors

Growing evidence from numerous preclinical studies indicates that pharmacological targeting of the SUMO pathway may be an effective therapeutic strategy in certain cancers. However, clinical data of SUMO inhibition are scarce. To date, the SAE inhibitor TAK-981 shows the most promise for clinical purposes and is currently the only compound directly targeting SUMOylation that has progressed to clinical trials in patients (Table 3).

A phase I/II study is evaluating the safety, tolerability, and preliminary efficacy of TAK-981 in combination with the immunotherapy drug pembrolizumab, in patients with advanced or metastatic solid tumors (ClinicalTrials.gov identifier: NCT04381650). Another phase I/II study is determining the safety and efficacy of TAK-981 in combination with anti-CD38 monoclonal antibodies, mezagitamab or daratumumab/hyaluronidase-fihj, for the treatment of patients with relapsed or refractory multiple myeloma (NCT04776018). The safety, tolerability, and efficacy of TAK-981 in combination with the anti-CD20 antibody rituximab, is evaluated in a phase I/II study of participants with CD20-positive non-Hodgkin lymphoma (NCT04074330). Furthermore, the safety, tolerability, and preliminary efficacy of TAK-981 as a monotherapy is evaluated a phase I/II study in participants with advanced or metastatic solid tumors or relapsed/refractory non-Hodgkin lymphomas (NCT03648372). In addition, an early phase I (phase 0) intratumoral microdosing study is investigating TAK-981′s ability to activate innate immune effector cells within the tumor microenvironment in patients with localized or metastatic tumors of the head and neck (NCT04065555). The same study is also testing whether treatment with TAK-981 in combination with the immunotherapy drug avelumab or anti-EGFR antibody cetuximab, results in enhanced localized immune responses when compared to either immunotherapy alone.

Unfortunately, no other direct inhibitors of SUMO pathway components have progressed to clinical testing in patients. However, SENP1 is investigated as a potential therapeutic target in two observational ex vivo clinical trials. One study aims at determining the ability of a novel SENP1 inhibitor, senpPNA-R8, to silence SENP1 expression and penetrate into organotypic osteosarcoma cultures prepared from freshly collected patient samples (NCT03798587). Another prospective study is evaluating the expression levels of SENP1 in bone metastatic and non-metastatic mammary carcinomas to determine its potential use as a therapeutic target and prognostic marker in breast cancer (NCT04167605).

### 7.5. Potential Rational Combinations

Given the role of SUMO in anti-tumor immunity, TAK-981 is currently being evaluated in combination with immunotherapy drugs in clinical trials. Dysregulation of SUMO pathway can also promote resistance towards several anti-cancer drugs, such as the proteasome inhibitor bortezomib in MM, anti-estrogens in breast cancer and different chemotherapeutics. Docetaxel resistance in PCa can be triggered via overexpression of the SENP1-HK2 axis or overexpression of PIAS1, further emphasizing heterogeneity of SUMO-dysregulation in cancer [239,303]. Hypoxia-induced SUMOylation of self-renewal and pluripotency regulator OCT4 decreases its level in embryonal carcinoma cells and promotes cisplatin and bleomycin resistance that can be counteracted with the induction of SENP1 overexpression [304]. Furthermore, SENP1-mediated deSUMOylation and the activation of janus kinase 2 (JAK2) promotes platinum resistance in ovarian cancer, which can be overcome by pharmacological inhibition of SENP1 that sensitizes cancer cells to cisplatin [305]. Thus, the combining of SUMO pathway inhibitors with already-established chemotherapy drugs and other anti-cancer agents may result in the improved targeting of specific cancer types through drug synergism and combat resistance.

## 8. Conclusions and Future Perspectives

Accumulating evidence strongly implicates a central role for SUMOylation in some cancer types as described earlier in detail. Intriguingly, the inhibition of SUMOylation may also be a novel way to activate the immune response to fight cancer cells, making the pharmacological inhibition of SUMOylation an attractive therapeutic strategy for the treatment of cancer patients. In the future, more sophisticated preclinical models of different contexts—also with an intact tumor microenvironment—are needed to fully characterize the biological significance of the SUMO pathway in cancer and explore its full therapeutic potential. Moreover, more individualized analyses of pathway activity in cancer patients with different types of hematological malignancies and solid tumors are needed to reveal potential therapeutic vulnerabilities and characterize potentially important new substrates.

Given the reversible nature of SUMO modification, it is important to deeply consider the balance between the regulators of SUMOylation to understand the final net contribution to malignant phenotypes in different cancers. Possible hurdles include the SUMOylation-independent functions of E3 ligases or deSUMOylases that may complicate the interpretation of the data. SENPs are essential for the balance of SUMOylation and the deSUMOylation of substrates. Interestingly, the development of SENP inhibitors has yielded encouraging results, as several SENP inhibitors have been identified to date and show promising activity in preclinical experiments. However, no selective inhibitors of SENPs other than SENP1 and SENP2 or SUMO E3 ligation enzymes are currently available despite recent strides made in the development of SUMO pathway inhibitors, limiting the identification and validation of novel therapeutic strategies towards SUMO-dysregulated cancers. Thus, the development of selective novel SENP and SUMO E3 ligase inhibitors is required to unravel the full potential of targeting SUMO machinery in cancer treatments and would open many new avenues to be explored, for example by using patient-derived ex vivo tumor models in combination with SUMO-pathway activity measurements. Furthermore, bifunctional small molecules that induce proximity between target proteins may be developed to trigger proteasomal degradation of SUMO machinery components or SUMO modification of specific proteins [306].

More than 20 years of intensive basic research is finally beginning to bear fruit, as the first clinical trials are investigating the safety and activity of SUMO pathway inhibition in cancer patients. The results of the first clinical trials with SUMO pathway inhibition in cancer patients are awaited with great interest, and they will help set new directions for research in this field. Since its discovery, SUMO has now strongly moved to the forefront of translational cancer research.

## Figures and Tables

**Table 1:** Prognostic value of SUMO pathway components. More comprehensive list of studies with prognostic and expression data and all references are in Supplementary Table S1.

High Expression Associates with Poor Prognosis		High Expression Associates with Good Prognosis	
Cancer Type	Protein(s)	Cancer Type	Protein(s)
adrenocortical	PIAS3, PIAS4, SAE1, SAE2, SENP1, SENP3, SUMO1, SUMO2, SUMO4	bladder	Ubc9
breast	PC2, PIAS3, PIAS4, SAE1, SAE2, SENP5, SENP7L, SUMO1, SUMO2, SUMO3, Ubc9	breast	PC2, PIAS1, PIAS4
colorectal	SAE2, SENP1, SUMO1	cervical	PIAS3
gastric	PC2, PIAS2, SAE2, SUMO3, Ubc9	colorectal	PC2
glioma	SAE1, Ubc9	gastric	PIAS1, PIAS4
hepatocellular	PC2, PIAS2, PIAS3, PIAS4, SAE1, SAE2, SENP1, SENP3, SENP5, SENP6, SUMO2, Ubc9	glioma	PIAS3
leukemia	SAE1, SUMO3	leukemia	PIAS2, SENP5, SENP7
lung	PC2, SAE1, SAE2, SENP1, SUMO2/3, SUMO4, Ubc9	lung	PIAS3
melanoma (cutaneous)	SAE1	melanoma (cutaneous)	PIAS1, SENP5, SENP7
melanoma (uveal)	SAE1, SAE2, SUMO3	melanoma (uveal)	SENP2, Ubc9
mesothelioma	PIAS3, PIAS4, SAE1, SAE2, SENP1	mesothelioma	PC2, PIAS3, SENP2
multiple myeloma	Ubc9	ovarian	PIAS2
osteosarcoma	PC2, SENP3	pancreatic	SENP3
ovarian	SENP3, SENP5	pheochromocytoma and paraganglioma	Ubc9
pancreatic	SENP2, (SUMO1 and SUMO2/3 together), Ubc9	renal	PIAS1, PIAS2
prostate	PIAS1, SAE1, SENP1, SENP5, SUMO1, SUMO2	testicular germ cell	PIAS2
renal	PC2, PIAS3, RSUME, SAE1, SENP1, SENP3, SENP5, SUMO1, SUMO2, Ubc9	thymoma	PIAS4, SAE1, SAE2, SENP1, Ubc9
sarcoma	PC2, PIAS2, PIAS3, SENP6, SENP7		
thyroid	PIAS2, SAE1		
uterine corpus endometrial	PC2, SAE2, SENP2, SENP5, SUMO4		

**Table 2:** Preclinical development of inhibitors targeting SUMO pathway. IC50 = half maximal inhibitory concentration of SUMOylation or deSUMOylation.

Target	Inhibitor	Product Type	Activity	IC50 (μM)	Study
SAE1/2	Ginkgolic acid	Natural	In vivo and in vitro	3.0	[263]
	Anacardic acid	Natural	In vivo and in vitro	2.2	[263]
	Kerriamycin B	Natural	In vitro	11.7	[264]
	SUMO-AMSN and SUMO-AVSN	Semisynthetic	In vitro		[302]
	Compound 9	Synthetic	In vitro	13.4	[267]
	Compound 21	Synthetic	In vitro	14.4	[268]
	Pyrazole and thiazole urea containing compounds	Synthetic	In vitro	13.8–100	[269]
	Davidiin	Natural	In vitro	0.15	[265]
	Tannic acid	Natural	In vivo and in vitro	12.8	[266]
	ML-792	Synthetic	In vitro	0.003 (SUMO1), 0.011 (SUMO2)	[270]
	COH000	Synthetic	In vivo and in vitro	0.2	[271,272]
	ML-93	Synthetic	In vitro	0.037	[273]
	Ginkgolic acid derivatives	Synthetic	In vitro	5–50	[274]
	TAK-981	Synthetic	In vivo and in vitro	nM range	[275]
Ubc9	GSK145A	Synthetic	In vitro	12.5	[280]
	2-D08	Synthetic	In vitro	6.0	[281]
	Spectomycin B1	Natural	In vivo and in vitro	4.4	[279]
	SUBINs	Semisynthetic	In vitro	0.025	[283]
	Compound 2	Synthetic	In vitro	74	[282]
SENP1	Compound 38	Synthetic	In vitro	9.2	[287]
	Triptolide	Natural	In vivo and in vitro	0.071–0.076	[236]
	Compound J5	Synthetic	In vitro	2.385	[288]
	Compound 4 (GN6958)	Synthetic	In vitro	29.6	[289]
	Compound 13m	Synthetic	In vitro	3.5	[290]
	Momordin Ic (Mc)	Natural	In vivo and in vitro	15.37	[237]
	Compounds 6, 7 and 10	Synthetic	In vitro	3.7, 0.99, 7.5	[291]
SENP2	Compounds 69 and 117	Synthetic	In vitro	5.9, 3.7	[292]
	Ebselen	Synthetic	In vivo and In vitro	2.0	[293]
SENP3	URB597	Synthetic			[294]
SENP1/2/3/5/6/7	JCP666	Natural	In vitro	13.8 (SENP1), 7.0 (SENP2)	[295,296]
	VEA260	Synthetic	In vitro	7.1 (SENP1), 3.7 (SENP2)	[295,296]
	VEA499	Synthetic	In vitro	3.6 (SENP1), 0.25 (SENP2)	[295]
	Compound 1	Synthetic	In vitro	5–10 (SENP1), 5–10 (SENP2)	[297]
	Compound 3	Synthetic	In vitro	3.55 (SENP1), 2.98 (SENP2)	[298]
	Streptonigrin	Natural	In vitro	0.518 (SENP1), 6.919 (SENP2)	[299]
	SI2	Synthetic	In vitro	1.29 (SENP1), unknown (SENP2), unknown (SENP3)	[240]
	Benzothiophene-2-carboxamide derivatives	Synthetic	In vitro	0.76–35.8 (SENP1), 0.56–75.6 (SENP2), 2.4–100 (SENP5)	[300]
	SPI-01	Synthetic	In vitro	5.9 (SENP1), 2.9 (SENP2), 3.5 (SENP7)	[301]
	VEA561	Synthetic	In vitro	5.7 (SENP2), 4.2 (SENP6), 4.3 (SENP7)	[295]

**Table 3:** Ongoing clinical trials of SUMO E1 inhibitor TAK-981. Data collected from clinicaltrials.gov (2.8.2021). Abbreviations: NCT# national clinical trial identifier number.

Intervention	Condition	Status	Estimated Enrollment	Phase	NCT#
TAK-981 and Pembrolizumab	Advanced or metastatic solid tumors	Recruiting	242	I/II	NCT04381650
TAK-981 and Mezagitamab and Daratumumab/hyaluronidase-fihj	Relapsed and/or refractory multiple myeloma	Recruiting	81	I/II	NCT04776018
TAK-981 and Rituximab	Non-Hodgkin lymphoma	Recruiting	130	I/II	NCT04074330
TAK-981	Advanced or metastatic solid tumors, Relapsed/refractory Non-Hodgkin lymphomas	Recruiting	202	I/II	NCT03648372
TAK-981 and Cetuximab and Avelumab	Head and neck cancer	Recruiting	12	0/I	NCT04065555

## Supplementary Materials

The following are available online at https://www.mdpi.com/article/10.3390/cancers13174402/s1, Table S1: “Expression and prognostic significance of SUMO machinery components in cancer” and supplementary references.
