# Supplementary material for: Therapeutic Potential of Targeting the SUMO Pathway in Cancer
Source: Cancers (Basel). 2021 Aug 31;13(17):4402. doi: 10.3390/cancers13174402 (PMC8431684; doi:10.3390/cancers13174402)
Supplement: Supplementary file 1 [file cancers-13-04402-s001.zip › cancers-1350973-supplementary.pdf]

References for Supplementary Table S1

1. Abbas, R.; McColl, K.S.; Kresak, A.; Yang, M.; Chen, Y.; Fu, P.; Wildey, G.; Dowlati, A. PIAS3 expression in squamous cell lung cancer is low and predicts overall survival. *Cancer Med.* **2015**, *4*, 325–332, doi:10.1002/cam4.372.
2. Agboola, A.; Musa, A.; Banjo, A.; Ayoade, B.; Deji-Agboola, M.; Nolan, C.; Rakha, E.; Ellis, I.; Green, A. PIASγ expression in relation to clinicopathological, tumour factors and survival in indigenous black breast cancer women. *J. Clin. Pathol.* **2013**, *67*, 301–306, doi:10.1136/jclinpath-2013-201658.
3. Agboola, A.; Musa, A.; Ayoade, B.; Banjo, A.; Anunobi, C.; Rakha, E.; Nolan, C.; Ellis, I.; Green, A. Clinicopathological and molecular significance of Sumolyation marker (ubiquitin conjugating enzyme 9 (UBC9)) expression in breast cancer of black women. *Pathol. - Res. Pr.* **2013**, *210*, 10–17, doi:10.1016/j.prp.2013.09.011.
4. Alshareeda, A.T.; Negm, O.H.; Green, A.; Nolan, C.; Tighe, P.; Albarakati, N.; Sultana, R.; Madhusudan, S.; Ellis, I.; Rakha, E.A. SUMOylation proteins in breast cancer. *Breast Cancer Res. Treat.* **2014**, *144*, 519–530, doi:10.1007/s10549-014-2897-7.
5. Bawa-Khalfe, T.; Cheng, J.; Lin, S.-H.; Ittmann, M.M.; Yeh, E.T.H. SENP1 Induces Prostatic Intraepithelial Neoplasia through Multiple Mechanisms. *J. Biol. Chem.* **2010**, *285*, 25859–25866, doi:10.1074/jbc.m110.134874.
6. Bawa-Khalfe, T.; Lu, L.-S.; Zuo, Y.; Huang, C.; Dere, R.; Lin, F.-M.; Yeh, E.T.H. Differential expression of SUMO-specific protease 7 variants regulates epithelial-mesenchymal transition. *Proc. Natl. Acad. Sci.* **2012**, *109*, 17466–17471, doi:10.1073/pnas.1209378109.
7. Biederstädt, A.; Hassan, Z.; Schneeweis, C.; Schick, M.; Schneider, L.; Muckenhuber, A.; Hong, Y.; Siegers, G.; Nilsson, L.; Wirth, M.; et al. SUMO pathway inhibition targets an aggressive pancreatic cancer subtype. *Gut* **2020**, *69*, 1472–1482, doi:10.1136/gutjnl-2018-317856.
8. Bouchard, D.M.; Matunis, M.J. A cellular and bioinformatics analysis of the SENP1 SUMO isopeptidase in pancreatic cancer. *J. Gastrointest. Oncol.* **2019**, *10*, 821–830, doi:10.21037/jgo.2019.05.09.
9. Brantley, E.C.; Nabors, L.; Gillespie, G.Y.; Choi, Y.-H.; Palmer, C.; Harrison, K.; Roarty, K.; Benveniste, E.N. Loss of Protein Inhibitors of Activated STAT-3 Expression in Glioblastoma Multiforme Tumors: Implications for STAT-3 Activation and Gene Expression. *Clin. Cancer Res.* **2008**, *14*, 4694–4704, doi:10.1158/1078-0432.ccr-08-0618.
10. Brems-Eskildsen, A.S.; Zieger, K.; Toldbod, H.; Holcomb, C.; Higuchi, R.; Mansilla, F.; Munksgaard, P.P.; Borre, M.; Ørntoft, T.F.; Dyrskjøt, L. Prediction and diagnosis of bladder cancer recurrence based on urinary content of hTERT, SENP1, PPP1CA, and MCM5 transcripts. *BMC Cancer* **2010**, *10*, 646, doi:10.1186/1471-2407-10-646.
11. Burdelski, C.; Menan, D.; Tsourlakis, M.C.; Kluth, M.; Hube-Magg, C.; Melling, N.; Minner, S.; Koop, C.; Graefen, M.; Heinzer, H.; et al. The prognostic value of SUMO1/Sentrin specific peptidase 1 (SENP1) in prostate cancer is limited to ERG-fusion positive tumors lacking PTEN deletion. *BMC Cancer* **2015**, *15*, 538, doi:10.1186/s12885-015-1555-8.
12. Cashman, R.; Cohen, H.; Ben-Hamo, R.; Zilberberg, A.; Efroni, S. SENP5 mediates breast cancer invasion via a TGFβRI SUMOylation cascade. *Oncotarget* **2014**, *5*, 1071–1082, doi:10.18632/oncotarget.1783.
13. Chanda, A.; Chan, A.; Deng, L.; Kornaga, E.N.; Enwere, E.K.; Morris, D.G.; Bonni, S. Identification of the SUMO E3 ligase PIAS1 as a potential survival biomarker in breast cancer. *PLOS ONE* **2017**, *12*, e0177639, doi:10.1371/journal.pone.0177639.
14. Chen, P.; Zhao, D.; Sun, Y.; Huang, L.; Zhang, S.; Yuan, Y. Protein inhibitor of activated STAT-1 is downregulated in gastric cancer tissue and involved in cell metastasis. *Oncol. Rep.* **2012**, *28*, 2149–2155, doi:10.3892/or.2012.2030.
15. Chen, S.-F.; Gong, C.; Luo, M.; Yao, H.-R.; Zeng, Y.-J.; Su, F.-X. Ubc9 expression predicts chemoresistance in breast cancer. *Chin. J. Cancer* **2011**, *30*, 638–644, doi:10.5732/cjc.011.10084.
16. Chen, X.; Wang, S.; Liang, X.; Liang, H.; Wang, T.; Wu, S.; Qiu, Z.; Zhan, R.; Xu, Z. SENP2 exerts an anti-tumor effect on chronic lymphocytic leukemia cells through the inhibition of the Notch and NF-κB signaling pathways. *Int. J. Oncol.* **2018**, *54*, 455–466, doi:10.3892/ijo.2018.4635.
17. Cheng, J.; Bawa, T.; Lee, P.; Gong, L.; Yeh, E.T. Role of Desumoylation in the Development of Prostate Cancer. *Neoplasia* **2006**, *8*, 667–676, doi:10.1593/neo.06445.
18. Cheng, J.; Su, M.; Jin, Y.; Xi, Q.; Deng, Y.; Chen, J.; Wang, W.; Chen, Y.; Chen, L.; Shi, N.; et al. Upregulation of SENP3/SMT3IP1 promotes epithelial ovarian cancer progression and forecasts poor prognosis. *Tumor Biol.* **2017**, *39*, doi:10.1177/1010428317694543.
19. Chien, W.; Lee, K.L.; Ding, L.-W.; Wuensche, P.; Kato, H.; Doan, N.B.; Poellinger, L.; Said, J.W.; Koeffler, H.P. PIAS4 is an activator of hypoxia signalling via VHL suppression during growth of pancreatic cancer cells. *Br. J. Cancer* **2013**, *109*, 1795–1804, doi:10.1038/bjc.2013.531.

20. Collaud, S.; Tischler, V.; Atanassoff, A.; Wiedl, T.; Komminoth, P.; Oehlschlegel, C.; Weder, W.; Soltermann, A. Lung neuroendocrine tumors: correlation of ubiquitinylation and sumoylation with nucleo-cytosolic partitioning of PTEN. *BMC Cancer* **2015**, *15*, 74, doi:10.1186/s12885-015-1084-5.
21. Coppola, D.; Parikh, V.; Boulware, D.; Blanck, G. Substantially reduced expression of PIAS1 is associated with colon cancer development. *Journal of Cancer Research and Clinical Oncology* **2009**, *135*, 1287–1291, doi:10.1007/s00432-009-0570-z.
22. Dabir, S.; Kluge, A.; Kresak, A.; Yang, M.; Fu, P.; Groner, B.; Wildey, G.; Dowlati, A. Low PIAS3 Expression in Malignant Mesothelioma Is Associated with Increased STAT3 Activation and Poor Patient Survival. *Clin. Cancer Res.* **2014**, *20*, 5124–5132, doi:10.1158/1078-0432.ccr-14-1233.
23. Sun; Ding, X.; Sun, J.; Wang, L.; Li, G.; Shen, Y.; Zhou, X.; Chen, W. Overexpression of SENP5 in oral squamous cell carcinoma and its association with differentiation. *Oncol. Rep.* **1994**, *20*, 1041–1045, doi:10.3892/or_00000107.
24. Diniz, M.G.; Silva, J.D.F.C.; De Souza, F.T.A.; Pereira, N.B.; Gomes, C.; Gomez, R.S. Association between cell cycle gene transcription and tumor size in oral squamous cell carcinoma. *Tumor Biol.* **2015**, *36*, 9717–9722, doi:10.1007/s13277-015-3735-1.
25. Dong, B.; Gao, Y.; Kang, X.; Gao, H.; Zhang, J.; Guo, H.; You, M.J.; Xue, W.; Cheng, J.; Huang, Y. SENP1 promotes proliferation of clear cell renal cell carcinoma through activation of glycolysis. *Oncotarget* **2016**, *7*, 80435–80449, doi:10.18632/oncotarget.12606.
26. Driscoll, J.J.; Pelluru, D.; Lefkimmiatis, K.; Fulciniti, M.; Prabhala, R.H.; Greipp, P.R.; Barlogie, B.; Tai, Y.-T.; Anderson, K.C.; Shaughnessy, J.D.; et al. The sumoylation pathway is dysregulated in multiple myeloma and is associated with adverse patient outcome. *Blood* **2010**, *115*, 2827–2834, doi:10.1182/blood-2009-03-211045.
27. Guo, W.-H.; Yuan, L.-H.; Xiao, Z.-H.; Liu, D.; Zhang, J.-X. Overexpression of SUMO-1 in hepatocellular carcinoma: a latent target for diagnosis and therapy of hepatoma. *Journal of Cancer Research and Clinical Oncology* **2010**, *137*, 533–541, doi:10.1007/s00432-010-0920-x.
28. Han, Y.; Huang, C.; Sun, X.; Xiang, B.; Wang, M.; Yeh, E.T.H.; Chen, Y.; Li, H.; Shi, G.; Cang, H.; et al. SENP3-mediated De-conjugation of SUMO2/3 from Promyelocytic Leukemia Is Correlated with Accelerated Cell Proliferation under Mild Oxidative Stress. *J. Biol. Chem.* **2010**, *285*, 12906–12915, doi:10.1074/jbc.m109.071431.
29. He, P.; Sun, X.; Cheng, H.; Zou, Y.; Wang, Q.; Zhou, C.; Liu, W.; Hao, Y.; Meng, X. UBA2 promotes proliferation of colorectal cancer. *Mol. Med. Rep.* **2018**, *18*, 5552–5562, doi:10.3892/mmr.2018.9613.
30. Hoefer, J.; Schäfer, G.; Klocker, H.; Erb, H.; Mills, I.G.; Hengst, L.; Puhr, M.; Culig, Z. PIAS1 Is Increased in Human Prostate Cancer and Enhances Proliferation through Inhibition of p21. *Am. J. Pathol.* **2012**, *180*, 2097–2107, doi:10.1016/j.ajpath.2012.01.026.
31. Hu, C.; Zhang, Q.; Tang, Q.; Zhou, H.; Liu, W.; Huang, J.; Liu, Y.; Wang, Q.; Zhang, J.; Zhou, M.; et al. CBX4 promotes the proliferation and metastasis via regulating BMI-1 in lung cancer. *J. Cell. Mol. Med.* **2020**, *24*, 618–631, doi:10.1111/jcmm.14771.
32. Huang, F.-J.; Dang, J.-Q.; Zhang, S.; Cheng, Z.-Y. Circular RNA hsa_circ_0008039 promotes proliferation, migration and invasion of breast cancer cells through upregulating CBX4 via sponging miR-515-5p. *European Review for Medical and Pharmacological Sciences* **2020**, *24*, 1887–1898.
33. Hung, P.-F.; Hong, T.-M.; Chang, C.-C.; Hung, C.-L.; Hsu, Y.-L.; Chang, Y.-L.; Wu, C.-T.; Chang, G.-C.; Chan, N.-L.; Yu, S.-L.; et al. Hypoxia-induced Slug SUMOylation enhances lung cancer metastasis. *J. Exp. Clin. Cancer Res.* **2019**, *38*, 5, doi:10.1186/s13046-018-0996-8.
34. Inamura, K.; Shimoji, T.; Ninomiya, H.; Hiramatsu, M.; Okui, M.; Satoh, Y.; Okumura, S.; Nakagawa, K.; Noda, T.; Fukayama, M.; et al. A metastatic signature in entire lung adenocarcinomas irrespective of morphological heterogeneity. *Hum. Pathol.* **2007**, *38*, 702–709, doi:10.1016/j.humpath.2006.11.019.
35. Ji, P.; Liang, S.; Li, P.; Xie, C.; Li, J.; Zhang, K.; Zheng, X.; Feng, M.; Li, Q.; Jiao, H.; et al. Speckle-type POZ protein suppresses hepatocellular carcinoma cell migration and invasion via ubiquitin-dependent proteolysis of SUMO1/sentrin specific peptidase 7. *Biochem. Biophys. Res. Commun.* **2018**, *502*, 30–42, doi:10.1016/j.bbrc.2018.05.115.
36. Jiang, B.; Fan, X.; Zhang, D.; Liu, H.; Fan, C. Identifying UBA2 as a proliferation and cell cycle regulator in lung cancer A549 cells. *J. Cell. Biochem.* **2019**, *120*, 12752–12761, doi:10.1002/jcb.28543.
37. Jiao, D.; Wu, M.; Ji, L.; Liu, F.; Liu, Y. MicroRNA-186 Suppresses Cell Proliferation and Metastasis Through Targeting Sentrin-Specific Protease 1 in Renal Cell Carcinoma. *Oncol. Res. Featur. Preclin. Clin. Cancer Ther.* **2018**, *26*, 249–259, doi:10.3727/096504017x14953948675430.
38. Jiao, H.-K.; Xu, Y.; Li, J.; Wang, W.; Mei, Z.; Long, X.-D.; Chen, G.-Q. Prognostic significance of Cbx4 expression and its beneficial effect for transarterial chemoembolization in hepatocellular carcinoma. *Cell Death Dis.* **2015**, *6*, e1689, doi:10.1038/cddis.2015.57.

39. Jin, Y.; Liu, Y.; Zhang, J.; Huang, W.; Jiang, H.; Hou, Y.; Xu, C.; Zhai, C.; Gao, X.; Wang, S.; et al. The Expression of miR-375 Is Associated with Carcinogenesis in Three Subtypes of Lung Cancer. *PLOS ONE* **2015**, *10*, e0144187, doi:10.1371/journal.pone.0144187.
40. Jin, Z.-L.; Pei, H.; Xu, Y.-H.; Yu, J.; Deng, T. The SUMO-specific protease SENP5 controls DNA damage response and promotes tumorigenesis in hepatocellular carcinoma. *Eur. Rev. Med Pharmacol. Sci.* **2016**, *20*, 3566–73.
41. Joseph, N.A.; Chiou, S.-H.; Lung, Z.; Yang, C.-L.; Lin, T.-Y.; Chang, H.-W.; Sun, H.S.; Gupta, S.K.; Yen, L.; Wang, S.-D.; et al. The role of HGF-MET pathway and CCDC66 cirRNA expression in EGFR resistance and epithelial-to-mesenchymal transition of lung adenocarcinoma cells. *J. Hematol. Oncol.* **2018**, *11*, 1–14, doi:10.1186/s13045-018-0557-9.
42. Kessler, J.D.; Kahle, K.T.; Sun, T.; Meerbrey, K.L.; Schlabach, M.R.; Schmitt, E.M.; Skinner, S.O.; Xu, Q.; Li, M.Z.; Hartman, J.; et al. A SUMOylation-Dependent Transcriptional Subprogram Is Required for Myc-Driven Tumorigenesis. *Sci.* **2012**, *335*, 348–353, doi:10.1126/science.1212728.
43. Kluge, A.; Dabir, S.; Vlassenbroeck, I.; Eisenberg, R.; Dowlati, A. Protein inhibitor of activated STAT3 expression in lung cancer. *Mol. Oncol.* **2011**, *5*, 256–264, doi:10.1016/j.molonc.2011.03.004.
44. Lee, J.-S.; Chu, I.-S.; Heo, J.; Calvisi, D.F.; Sun, Z.; Roskams, T.; Durnez, A.; Demetris, A.J.; Thorgeirsson, S.S. Classification and prediction of survival in hepatocellular carcinoma by gene expression profiling. *Hepatol.* **2004**, *40*, 667–676, doi:10.1002/hep.20375.
45. Li, H.; Niu, H.; Peng, Y.; Wang, J.; He, P. Ubc9 promotes invasion and metastasis of lung cancer cells. *Oncol. Rep.* **2013**, *29*, 1588–1594, doi:10.3892/or.2013.2268.
46. Li, J.; Xu, Y.; Long, X.-D.; Wang, W.; Jiao, H.-K.; Mei, Z.; Yin, Q.-Q.; Ma, L.-N.; Zhou, A.-W.; Wang, L.-S.; et al. Cbx4 Governs HIF-1α to Potentiate Angiogenesis of Hepatocellular Carcinoma by Its SUMO E3 Ligase Activity. *Cancer Cell* **2014**, *25*, 118–131, doi:10.1016/j.ccr.2013.12.008.
47. Li, J.; Sun, X.; He, P.; Liu, W.-Q.; Zou, Y.-B.; Wang, Q.; Meng, X.-W. Ubiquitin-like modifier activating enzyme 2 promotes cell migration and invasion through Wnt/β-catenin signaling in gastric cancer. *World J. Gastroenterol.* **2018**, *24*, 4773–4786, doi:10.3748/wjg.v24.i42.4773.
48. Li, P.; Yu, X.; Ge, K.; Melamed, J.; Roeder, R.G.; Wang, Z. Heterogeneous Expression and Functions of Androgen Receptor Co-Factors in Primary Prostate Cancer. *Am. J. Pathol.* **2002**, *161*, 1467–1474, doi:10.1016/s0002-9440(10)64422-7.
49. Li, T.; Huang, S.; Dong, M.; Gui, Y.; Wu, D. Prognostic impact of SUMO-specific protease 1 (SENP1) in prostate cancer patients undergoing radical prostatectomy. *Urol. Oncol. Semin. Orig. Investig.* **2013**, *31*, 1539–1545, doi:10.1016/j.urolonc.2012.03.007.
50. Li, X.; Gou, J.; Li, H.; Yang, X. Bioinformatic analysis of the expression and prognostic value of chromobox family proteins in human breast cancer. *Sci. Rep.* **2020**, *10*, 1–11, doi:10.1038/s41598-020-74792-5.
51. Li, X.; Meng, Y. SUMOylation Regulator-Related Molecules Can Be Used as Prognostic Biomarkers for Glioblastoma. *Front. Cell Dev. Biol.* **2021**, *9*, 689, doi:10.3389/fcell.2021.658856.
52. Liang, J.-X.; Gao, W.; Zeng, X.-W.; Cheng, G.-P.; Cai, L.; Tao, K.-Y.; Yang, X. SUMO4 small interfering RNA attenuates invasion and migration via the JAK2/STAT3 pathway in non-small cell lung cancer cells. *Oncol. Lett.* **2020**, *20*, 1, doi:10.3892/ol.2020.12088.
53. Lin, F.-M.; Kumar, S.; Ren, J.; Karami, S.; Bahnassy, S.; Li, Y.; Zheng, X.; Wang, J.; Bawa-Khalfe, T. SUMOylation of HP1α supports association with ncRNA to define responsiveness of breast cancer cells to chemotherapy. *Oncotarget* **2016**, *7*, 30336–30349, doi:10.18632/oncotarget.8733.
54. Lin, K.; Zhu, J.; Hu, C.; Bu, F.; Luo, C.; Zhu, X.; Zhu, Z. Comprehensive analysis of the prognosis for chromobox family in gastric cancer. *J. Gastrointest. Oncol.* **2020**, *11*, 932–951, doi:10.21037/jgo-20-208.
55. Lin, Y.; Wang, Q.; Lin, Y.; Jiang, M.; Xiao, H.; Zhang, J.; Guo, R.; Kang, S.; Lin, Y.; Song, C. An immunohistochemical panel of three small ubiquitin-like modifier genes predicts outcomes of patients with triple-negative breast cancer. *Gland. Surg.* **2021**, *10*, 1067–1084, doi:10.21037/gs-21-37.
56. Linja, M.J.; Porkka, K.P.; Kang, Z.; Savinainen, K.J.; Jänne, O.A.; Tammela, T.L.J.; Vessella, R.L.; Palvimo, J.; Visakorpi, T. Expression of Androgen Receptor Coregulators in Prostate Cancer. *Clin. Cancer Res.* **2004**, *10*, 1032–1040, doi:10.1158/1078-0432.ccr-0990-3.
57. Liu, B.; Tahk, S.; Yee, K.M.; Yang, R.; Yang, Y.; Mackie, R.; Hsu, C.; Chernishof, V.; O'Brien, N.; Jin, Y.; et al. PIAS1 Regulates Breast Tumorigenesis through Selective Epigenetic Gene Silencing. *PLOS ONE* **2014**, *9*, e89464, doi:10.1371/journal.pone.0089464.
58. Liu, F.; Li, L.; Li, Y.; Ma, X.; Bian, X.; Liu, X.; Wang, G.; Zhang, D. Overexpression of SENP1 reduces the stemness capacity of osteosarcoma stem cells and increases their sensitivity to HSVtk/GCV. *Int. J. Oncol.* **2018**, *53*, 2010–2020, doi:10.3892/ijo.2018.4537.

59. Liu, K.; Zhang, J.; Wang, H. Small ubiquitin-like modifier/sentrin-specific peptidase 1 associates with chemotherapy and is a risk factor for poor prognosis of non-small cell lung cancer. *J. Clin. Lab. Anal.* **2018**, *32*, e22611, doi:10.1002/jcla.22611.
60. Liu, L.-H.; Li, H.; Cheng, X.-X.; Kong, Q.-Y.; Chen, X.-Y.; Wu, M.-L.; Li, Y.; Liu, J.; Li, C. Correlative analyses of the expression levels of PIAS3, p-SHP2, SOCS1 and SOCS3 with STAT3 activation in human astrocytomas. *Mol. Med. Rep.* **2016**, *15*, 847–852, doi:10.3892/mmr.2016.6079.
61. Liu, L.-M.; Yan, M.-G.; Yang, D.-H.; Sun, W.-W.; Zhang, J.-X. PIAS3 expression in human gastric carcinoma and its adjacent non-tumor tissues. *Clin. Res. Hepatol. Gastroenterol.* **2011**, *35*, 393–398, doi:10.1016/j.clinre.2010.12.002.
62. Liu, Q.; Zhou, B.; Liao, R.; Zhou, X.; Yan, X. PIAS4, upregulated in hepatocellular carcinoma, promotes tumorigenicity and metastasis. *J. Cell. Biochem.* **2020**, *121*, 3372–3381, doi:10.1002/jcb.29610.
63. Liu, X.; Xu, Y.; Pang, Z.; Guo, F.; Qin, Q.; Yin, T.; Sang, Y.; Feng, C.; Li, X.; Jiang, L.; et al. Knockdown of SUMO-activating enzyme subunit 2 (SAE2) suppresses cancer malignancy and enhances chemotherapy sensitivity in small cell lung cancer. *J. Hematol. Oncol.* **2015**, *8*, 67, doi:10.1186/s13045-015-0164-y.
64. Ma, C.; Wu, B.; Huang, X.; Yuan, Z.; Nong, K.; Dong, B.; Bai, Y.; Zhu, H.; Wang, W.; Ai, K. SUMO-specific protease 1 regulates pancreatic cancer cell proliferation and invasion by targeting MMP-9. *Tumor Biol.* **2014**, *35*, 12729–12735, doi:10.1007/s13277-014-2598-1.
65. Mattoscio, D.; Casadio, C.; Fumagalli, M.; Sideri, M.; Chiocca, S. The SUMO conjugating enzyme UBC9 as a biomarker for cervical HPV infections. *ecancermedicalscience* **2015**, *9*, doi:10.3332/ecancer.2015.534.
66. Mattoscio, D.; Casadio, C.; Miccolo, C.; Maffini, F.; Raimondi, A.; Tacchetti, C.; Gheit, T.; Tagliabue, M.; Galimberti, V.E.; De Lorenzi, F.; et al. Autophagy regulates UBC9 levels during viral-mediated tumorigenesis. *PLOS Pathog.* **2017**, *13*, e1006262, doi:10.1371/journal.ppat.1006262.
67. McHale, K.; E Tomaszewski, J.; Puthiyaveettil, R.; A LiVolsi, V.; Clevenger, C.V. Altered expression of prolactin receptor-associated signaling proteins in human breast carcinoma. *Mod. Pathol.* **2008**, *21*, 565–571, doi:10.1038/modpathol.2008.7.
68. Meng, R.; Fang, J.; Yu, Y.; Hou, L.K.; Chi, J.R.; Chen, A.X.; Zhao, Y.; Cao, X.C. miR-129-5p suppresses breast cancer proliferation by targeting CBX4. *Neoplasma* **2018**, *65*, 572–578, doi:10.4149/neo_2018_170814n530.
69. Mo, Y.-Y.; Yu, Y.; Theodosiou, E.; Ee, P.L.R.; Beck, W.T. A role for Ubc9 in tumorigenesis. *Oncogene* **2005**, *24*, 2677–2683, doi:10.1038/sj.onc.1208210.
70. Mooney, S.M.; Grande, J.P.; Salisbury, J.; Janknecht, R. Sumoylation of p68 and p72 RNA Helicases Affects Protein Stability and Transactivation Potential. *Biochem.* **2010**, *49*, 1–10, doi:10.1021/bi901263m.
71. Moschos, S.J.; Smith, A.P.; Mandic, M.; Athanassiou, C.; Watson-Hurst, K.; Jukic, D.M.; Edington, H.D.; Kirkwood, J.M.; Becker, D. SAGE and antibody array analysis of melanoma-infiltrated lymph nodes: identification of Ubc9 as an important molecule in advanced-stage melanomas. *Oncogene* **2007**, *26*, 4216–4225, doi:10.1038/sj.onc.1210216.
72. Moschos, S.J.; Jukic, D.M.; Athanassiou, C.; Bhargava, R.; Dacic, S.; Wang, X.; Kuan, S.-F.; Fayewicz, S.L.; Galambos, C.; Acquafondata, M.; et al. Expression analysis of Ubc9, the single small ubiquitin-like modifier (SUMO) E2 conjugating enzyme, in normal and malignant tissues. *Hum. Pathol.* **2010**, *41*, 1286–1298, doi:10.1016/j.humpath.2010.02.007.
73. Mu, J.; Zuo, Y.; Yang, W.; Chen, Z.; Liu, Z.; Tu, J.; Li, Y.; Yuan, Z.; Cheng, J.; He, J. Over-expression of small ubiquitin-like modifier proteases 1 predicts chemo-sensitivity and poor survival in non-small cell lung cancer. *Chin. Med J.* **2014**, *127*, 4060–4065.
74. Ni, R.S.; Shen, X.; Qian, X.; Yu, C.; Wu, H.; Gao, X. Detection of differentially expressed genes and association with clinicopathological features in laryngeal squamous cell carcinoma. *Oncol. Lett.* **2012**, *4*, 1354–1360, doi:10.3892/ol.2012.920.
75. Nomair, A.M.; Ahmed, S.S.; Nomeir, H.M.; El Mansy, H.; Mohammed, A.F. The role of protein inhibitor of activated STAT3 and miRNA-18a expressions in breast cancer. *Egypt. J. Med Hum. Genet.* **2019**, *20*, 1–9, doi:10.1186/s43042-019-0021-6.
76. Alves, M.G.O.; Delgado, A.D.M.; Balducci, I.; Carvalho, Y.R.; Cavalcante, A.S.R.; Almeida, J.D. Study of MDM2 and SUMO-1 expression in actinic cheilitis and lip cancer. *Arch. Dermatol. Res.* **2014**, *306*, 837–841, doi:10.1007/s00403-014-1500-8.
77. Ong, J.; Bamodu, O.; Khang, N.; Lin, Y.-K.; Yeh, C.-T.; Lee, W.-H.; Cherng, Y.-G. SUMO-Activating Enzyme Subunit 1 (SAE1) Is a Promising Diagnostic Cancer Metabolism Biomarker of Hepatocellular Carcinoma. *Cells* **2021**, *10*, 178, doi:10.3390/cells10010178.
78. Pastuszak-Lewandoska, D.; Domanska, D.; Czarnecka, K.H.; Kordiak, J.; Migdalska-Sęk, M.; Nawrot, E.; Kiszałkiewicz, J.; Antczak, A.; Górski, P.; Brzeziańska, E. Expression of STAT5, COX-2 and PIAS3 in Correlation with NSCLC Histhopathological Features. *PLOS ONE* **2014**, *9*, e104265, doi:10.1371/journal.pone.0104265.

79. Pei, H.; Chen, L.; Liao, Q.; Wang, K.; Chen, S.; Liu, Z.; Zhang, Z. SUMO-specific protease 2 (SENP2) functions as a tumor suppressor in osteosarcoma via SOX9 degradation. *Exp. Ther. Med.* **2018**, *16*, 5359–5365, doi:10.3892/etm.2018.6838.
80. Polimeno, L.; Francavilla, A.; Piscitelli, D.; Fiore, M.G.; Polimeno, R.; Topi, S.; Haxhirexha, K.; Ballini, A.; Daniele, A.; Santacroce, L. The role of PIAS3, p-STAT3 and ALR in colorectal cancer: new translational molecular features for an old disease. *European Review for Medical and Pharmacological Sciences* **2020**, *24*, 10496–10511.
81. Puhr, M.; Hoefer, J.; Neuwirt, H.; Eder, I.E.; Kern, J.; Schäfer, G.; Geley, S.; Heidegger, I.; Klocker, H.; Culig, Z. PIAS1 is a crucial factor for prostate cancer cell survival and a valid target in docetaxel resistant cells. *Oncotarget* **2014**, *5*, 12043–12056, doi:10.18632/oncotarget.2658.
82. Puhr, M.; Hoefer, J.; Eigentler, A.; Dietrich, D.; Van Leenders, G.; Uhl, B.; Hoogland, M.; Handle, F.; Schlick, B.; Neuwirt, H.; et al. PIAS1 is a determinant of poor survival and acts as a positive feedback regulator of AR signaling through enhanced AR stabilization in prostate cancer. *Oncogene* **2015**, *35*, 2322–2332, doi:10.1038/onc.2015.292.
83. Qian, J.; Luo, Y.; Gu, X.; Wang, X. Inhibition of SENP6-Induced Radiosensitization of Human Hepatocellular Carcinoma Cells by Blocking Radiation-Induced NF-κB Activation. *Cancer Biotherapy Radiopharm.* **2013**, *28*, 196–200, doi:10.1089/cbr.2012.1288.
84. Qu, D.; Yang, Y.; Huang, X. miR-199a-5p promotes proliferation and metastasis and epithelial-mesenchymal transition through targeting PIAS3 in cervical carcinoma. *J. Cell. Biochem.* **2019**, *120*, 13562–13572, doi:10.1002/jcb.28631.
85. Ren, Y.-H.; Liu, K.-J.; Wang, M.; Yu, Y.-N.; Yang, K.; Chen, Q.; Yu, B.; Wang, W.; Li, Q.-W.; Wang, J.; et al. De-SUMOylation of FOXC2 by SENP3 promotes the epithelial-mesenchymal transition in gastric cancer cells. *Oncotarget* **2014**, *5*, 7093–7104, doi:10.18632/oncotarget.2197.
86. Renner, O.; Fominaya, J.; Alonso, S.; Blanco-Aparicio, C.; Leal, J.F.M.; Carnero, A. Mst1, RanBP2 and eIF4G are new markers for in vivo PI3K activation in murine and human prostate. *Carcinog.* **2007**, *28*, 1418–1425, doi:10.1093/carcin/bgm059.
87. Ronen, O.; Malone, J.P.; Kay, P.; Bivens, C.; Hall, K.; Paruchuri, L.P.; Mo, Y.-Y.; Robbins, K.T.; Ran, S. Expression of a novel marker, Ubc9, in squamous cell carcinoma of the head and neck. *Head Neck* **2009**, *31*, 845–855, doi:10.1002/hed.21048.
88. Shao, D.-F.; Wang, X.-H,; Li Z.-Y,; Xing X.-F.; Cheng, X.-J.; Guo, T.; Du, H.; Hu, Y., Dong, B.; Ding, N.; et al. High-level SAE2 promotes malignant phenotype and predicts outcome in gastric cancer. *Am. J. Cancer Res.* **2015**, 5(1), 140–54. Available at: https://www.ncbi.nlm.nih.gov/pmc/articles/PMC4300690/ (Accessed: 30 June 2021).
89. Shen, H.-J.; Zhu, H.-Y.; Yang, C.; Ji, F. SENP2 regulates hepatocellular carcinoma cell growth by modulating the stability of ?-catenin. *Asian Pac. J. Cancer Prev.* **2012**, *13*, 3583–3587, doi:10.7314/apjcp.2012.13.8.3583.
90. Soares, I.N.; Caetano, F.A.; Pinder, J.; Rodrigues, B.R.; Beraldo, F.H.; Ostapchenko, V.; Durette, C.; Pereira, G.; Lopes, M.; Queiroz-Hazarbassanov, N.; et al. Regulation of Stress-Inducible Phosphoprotein 1 Nuclear Retention by Protein Inhibitor of Activated STAT PIAS1. *Mol. Cell. Proteom.* **2013**, *12*, 3253–3270, doi:10.1074/mcp.m113.031005.
91. Stefanska, B.; Cheishvili, D.; Suderman, M.; Arakelian, A.; Huang, J.; Hallett, M.; Han, Z.-G.; Al-Mahtab, M.; Akbar, S.M.F.; Khan, W.A.; et al. Genome-Wide Study of Hypomethylated and Induced Genes in Patients with Liver Cancer Unravels Novel Anticancer Targets. *Clin. Cancer Res.* **2014**, *20*, 3118–3132, doi:10.1158/1078-0432.ccr-13-0283.
92. Sun, L.; Li, H.; Chen, J.; Iwasaki, Y.; Kubota, T.; Matsuoka, M.; Shen, A.; Chen, Q.; Xu, Y. PIASy mediates hypoxia-induced SIRT1 transcriptional repression and epithelial-to-mesenchymal transition in ovarian cancer cells. *J. Cell Sci.* **2013**, *126*, 3939–3947, doi:10.1242/jcs.127381.
93. Sun, Z.; Hu, S.; Luo, Q.; Ye, D.; Hu, D.; Chen, F. Overexpression of SENP3 in oral squamous cell carcinoma and its association with differentiation. *Oncol. Rep.* **2013**, *29*, 1701–1706, doi:10.3892/or.2013.2318.
94. Taheri, M.; Oskooei, V.K.; Ghafouri-Fard, S. Protein inhibitor of activated STAT genes are differentially expressed in breast tumor tissues. *Pers. Med.* **2019**, *16*, 277–285, doi:10.2217/pme-2018-0070.
95. Tan, M.-Y.; Mu, X.-Y.; Liu, B.; Wang, Y.; Bao, E.-D.; Qiu, J.-X.; Fan, Y. SUMO-Specific Protease 2 Suppresses Cell Migration and Invasion through Inhibiting the Expression of MMP13 in Bladder Cancer Cells. *Cell. Physiol. Biochem.* **2013**, *32*, 542–548, doi:10.1159/000354458.
96. Tao, Y.; Li, R.; Shen, C.; Li, J.; Zhang, Q.; Ma, Z.; Wang, F.; Wang, Z. SENP1 is a crucial promotor for hepatocellular carcinoma through deSUMOylation of UBE2T. *Aging* **2020**, *12*, 1563–1576, doi:10.18632/aging.102700.

97. Tedesco, L.; Elguero, B.; Pacin, D.G.; Senin, S.; Pollak, C.; Marchiñena, P.A.G.; Jurado, A.M.; Isola, M.; Labanca, M.J.; Palazzo, M.; et al. von Hippel-Lindau mutants in renal cell carcinoma are regulated by increased expression of RSUME. *Cell Death Dis.* **2019**, *10*, 1–13, doi:10.1038/s41419-019-1507-3.
98. Tomasi, M.L.; Tomasi, I.; Ramani, K.; Pascale, R.M.; Xu, J.; Giordano, P.; Mato, J.; Lu, S.C. S-adenosyl methionine regulates ubiquitin-conjugating enzyme 9 protein expression and sumoylation in murine liver and human cancers. *Hepatol.* **2012**, *56*, 982–993, doi:10.1002/hep.25701.
99. Tu, J.; Chen, Y.; Cai, L.; Xu, C.; Zhang, Y.; Chen, Y.; Zhang, C.; Zhao, J.; Cheng, J.; Xie, H.; et al. Functional Proteomics Study Reveals SUMOylation of TFII-I is Involved in Liver Cancer Cell Proliferation. *J. Proteome Res.* **2015**, *14*, 2385–2397, doi:10.1021/acs.jproteome.5b00062.
100. Tuccilli, C.; Baldini, E.; Sorrenti, S.; Di Gioia, C.; Bosco, D.; Ascoli, V.; Mian, C.; Barollo, S.; Rendina, R.; Coccaro, C.; et al. PAPILLARY THYROID CANCER IS CHARACTERIZED BY ALTERED EXPRESSION OF GENES INVOLVED IN THE SUMOYLATION PROCESS. *J. Boil. Regul. Homeost. agents* **2015**, *29*, 655–62.
101. Wang, B.; Tang, J.; Liao, D.; Wang, G.; Zhang, M.; Sang, Y.; Cao, J.; Wu, Y.; Zhang, R.; Li, S.; et al. Chromobox Homolog 4 Is Correlated with Prognosis and Tumor Cell Growth in Hepatocellular Carcinoma. *Ann. Surg. Oncol.* **2013**, *20*, 684–692, doi:10.1245/s10434-013-3171-7.
102. Wang, J.; Qian, J.; Hoeksema, M.D.; Zou, Y.; Espinosa, A.V.; Rahman, S.J.; Zhang, B.; Massion, P.P. Integrative Genomics Analysis Identifies Candidate Drivers at 3q26-29 Amplicon in Squamous Cell Carcinoma of the Lung. *Clin. Cancer Res.* **2013**, *19*, 5580–5590, doi:10.1158/1078-0432.ccr-13-0594.
103. Wang, J.; Ni, J.; Yi, S.; Song, D.; Ding, M. Protein inhibitor of activated STAT xα depresses cyclin D and cyclin D kinase, and contributes to the inhibition of osteosarcoma cell progression. *Mol. Med. Rep.* **2016**, *13*, 1645–1652, doi:10.3892/mmr.2015.4705.
104. Wang, L.; Banerjee, S. Differential PIAS3 expression in human malignancy. *Oncol. Rep.* **2004**, *11*, 1319–1324, doi:10.3892/or.11.6.1319.
105. Wang, Q.; Xia, N.; Li, T.; Xu, Y.; Zou, Y.; Zuo, Y.; Fan, Q.; Bawa-Khalfe, T.; Yeh, E.T.H.; Cheng, J. SUMO-specific protease 1 promotes prostate cancer progression and metastasis. *Oncogene* **2012**, *32*, 2493–2498, doi:10.1038/onc.2012.250.
106. Wang, R.-T.; Zhi, X.-Y.; Zhang, Y.; Zhang, J. Inhibition of SENP1 induces radiosensitization in lung cancer cells. *Exp. Ther. Med.* **2013**, *6*, 1054–1058, doi:10.3892/etm.2013.1259.
107. Wang, S.; Jiao, B.; Geng, S.; Ma, S.; Liang, Z.; Lu, S. Combined aberrant expression of microRNA-214 and UBC9 is an independent unfavorable prognostic factor for patients with gliomas. *Med Oncol.* **2013**, *31*, 767, doi:10.1007/s12032-013-0767-5.
108. Wang, X.-K.; Liao, X.-W.; Zhou, X.; Han, C.-Y.; Chen, Z.-J.; Yang, C.-K.; Huang, J.-L.; Wang, J.-Y.; Liu, J.-Q.; Huang, H.-S.; et al. Oncogene UBE2I enhances cellular invasion, migration and proliferation abilities via autophagy-related pathway resulting in poor prognosis in hepatocellular carcinoma. *American journal of cancer research. e-Century Publishing Corporation* 2020, *10*, 4178–4197.
109. Wang, X.; Li, L.; Wu, Y.; Zhang, R.; Zhang, M.; Liao, D.; Wang, G.; Qin, G.; Xu, R.-H.; Kang, T. CBX4 Suppresses Metastasis via Recruitment of HDAC3 to the Runx2 Promoter in Colorectal Carcinoma. *Cancer Res.* **2016**, *76*, 7277–7289, doi:10.1158/0008-5472.can-16-2100.
110. Wang, X.; Qin, G.; Liang, X.; Wang, W.; Wang, Z.; Liao, D.; Zhong, L.; Zhang, R.; Zeng, Y.-X.; Wu, Y.; et al. Targeting the CK1α/CBX4 axis for metastasis in osteosarcoma. *Nat. Commun.* **2020**, *11*, 1–14, doi:10.1038/s41467-020-14870-4.
111. Wang, Z.; Jin, J.; Zhang, J.; Wang, L.; Cao, J. Depletion of SENP1 suppresses the proliferation and invasion of triple-negative breast cancer cells. *Oncol. Rep.* **2016**, *36*, 2071–2078, doi:10.3892/or.2016.5036.
112. Wang, Z.; Fang, Z.; Chen, G.; Liu, B.; Xu, J.; Li, F.; Li, F.; Liu, H.; Zhang, H.; Sun, Y.; et al. Chromobox 4 facilitates tumorigenesis of lung adenocarcinoma through the Wnt/β-catenin pathway. *Neoplasia* **2021**, *23*, 222–233, doi:10.1016/j.neo.2020.12.005.
113. Wee, Y.; Liu, Y.; Lu, J.; Li, X.; Zhao, M. Identification of novel prognosis-related genes associated with cancer using integrative network analysis. *Sci. Rep.* **2018**, *8*, 1–11, doi:10.1038/s41598-018-21691-5.
114. Wei, J.; Costa, C.; Ding, Y.; Zou, Z.; Yu, L.; Sanchez, J.J.; Qian, X.; Chen, H.; Gimenez-Capitan, A.; Meng, F.; et al. mRNA Expression of BRCA1, PIAS1, and PIAS4 and Survival After Second-line Docetaxel in Advanced Gastric Cancer. *J. Natl. Cancer Inst.* **2011**, *103*, 1552–1556, doi:10.1093/jnci/djr326.
115. Wei, J.; Costa, C.; Shen, J.; Yu, L.; Sánchez, J.J.; Qian, X.; Sun, X.; Zou, Z.; Gimenez-Capitan, A.; Yue, G.; et al. Differential effect of MMSET mRNA levels on survival to first-line FOLFOX and second-line docetaxel in gastric cancer. *Br. J. Cancer* **2014**, *110*, 2662–2668, doi:10.1038/bjc.2014.231.
116. Wu, F.; Zhu, S.; Ding, Y.; Beck, W.T.; Mo, Y.-Y. MicroRNA-mediated Regulation of Ubc9 Expression in Cancer Cells. *Clin. Cancer Res.* **2009**, *15*, 1550–1557, doi:10.1158/1078-0432.CCR-08-0820.

117. Wu, G.; Xu, Y.; Ruan, N.; Li, J.; Lv, Q.; Zhang, Q.; Chen, Y.; Wang, Q.; Xia, Q.; Li, Q. Genetic alteration and clinical significance of SUMOylation regulators in multiple cancer types. *J. Cancer* **2020**, *11*, 6823–6833, doi:10.7150/jca.49042.
118. Wu, Y.; Tedesco, L.; Lucia, K.; Schlitter, A.M.; Garcia, J.M.; Esposito, I.; Auernhammer, C.J.; Theodoropoulou, M.; Arzt, E.; Renner, U.; et al. RSUME is implicated in tumorigenesis and metastasis of pancreatic neuroendocrine tumors. *Oncotarget* **2016**, *7*, 57878–57893, doi:10.18632/oncotarget.11081.
119. Xia, W.; Tian, H.; Cai, X.; Kong, H.; Fu, W.; Xing, W.; Wang, Y.; Zou, M.; Hu, Y.; Xu, D. Inhibition of SUMO-specific protease 1 induces apoptosis of astroglioma cells by regulating NF-κB/Akt pathways. *Gene* **2016**, *595*, 175–179, doi:10.1016/j.gene.2016.09.040.
120. Xiang-Ming, Y.; Zhi-Qiang, X.; Ting, Z.; Jian, W.; Jian, P.; Li-Qun, Y.; Ming-Cui, F.; Hong-Liang, X.; Xu, C.; Yun, Z. SENP1 regulates cell migration and invasion in neuroblastoma. *Biotechnol. Appl. Biochem.* **2016**, *63*, 435–440, doi:10.1002/bab.1375.
121. Xie, H.; Gu, Y.; Wang, W.; Wang, X.; Ye, X.; Xin, C.; Lu, M.; Reddy, B.A.; Shu, P. Silencing of SENP2 in Multiple Myeloma Induces Bortezomib Resistance by Activating NF-κB Through the Modulation of IκBα Sumoylation. *Sci. Rep.* **2020**, *10*, 766–10, doi:10.1038/s41598-020-57698-0.
122. Xu, Y.; Li, J.; Zuo, Y.; Deng, J.; Wang, L.-S.; Chen, G.-Q. SUMO-specific protease 1 regulates the in vitro and in vivo growth of colon cancer cells with the upregulated expression of CDK inhibitors. *Cancer Lett.* **2011**, *309*, 78–84, doi:10.1016/j.canlet.2011.05.019.
123. Yang, H.; Tang, Y.; Guo, W.; Du, Y.; Wang, Y.; Li, P.; Zang, W.; Yin, X.; Wang, H.; Chu, H.; et al. Up-regulation of microRNA-138 induce radiosensitization in lung cancer cells. *Tumor Biol.* **2014**, *35*, 6557–6565, doi:10.1007/s13277-014-1879-z.
124. Yang, H.; Gao, S.; Chen, J.; Lou, W. UBE2I promotes metastasis and correlates with poor prognosis in hepatocellular carcinoma. *Cancer Cell Int.* **2020**, *20*, 1–13, doi:10.1186/s12935-020-01311-x.
125. Yang, P.; Liu, Y.; Qi, Y.C.; Lian, Z.H. High SENP3 Expression Promotes Cell Migration, Invasion, and Proliferation by Modulating DNA Methylation of E-Cadherin in Osteosarcoma. *Technol. Cancer Res. Treat.* **2020**, *19*, doi:10.1177/1533033820956988.
126. Yang, S.-F.; Hou, M.-F.; Chen, F.-M.; Ou-Yang, F.; Wu, Y.-C.; Chai, C.-Y.; Yeh, Y.-T. Prognostic value of protein inhibitor of activated STAT3 in breast cancer patients receiving hormone therapy. *BMC Cancer* **2016**, *16*, 20, doi:10.1186/s12885-016-2063-1.
127. Yang, W.; Wang, L.; Roehn, G.; Pearlstein, R.D.; Ali-Osman, F.; Pan, H.; Goldbrunner, R.; Krantz, M.; Harms, C.; Paschen, W. Small ubiquitin-like modifier 1-3 is activated in human astrocytic brain tumors and is required for glioblastoma cell survival. *Cancer Sci.* **2012**, *104*, 70–77, doi:10.1111/cas.12047.
128. Yang, Y.; Liang, Z.; Xia, Z.; Wang, X.; Ma, Y.; Sheng, Z.; Gu, Q.; Shen, G.; Zhou, L.; Zhu, H.; et al. SAE1 promotes human glioma progression through activating AKT SUMOylation-mediated signaling pathways. *Cell Commun. Signal.* **2019**, *17*, 1–14, doi:10.1186/s12964-019-0392-9.
129. Zeng, J.-S.; Zhang, Z.-D.; Pei, L.; Bai, Z.-Z.; Yang, Y.; Yang, H.; Tian, Q.-H. CBX4 exhibits oncogenic activities in breast cancer via Notch1 signaling. *Int. J. Biochem. Cell Biol.* **2018**, *95*, 1–8, doi:10.1016/j.biocel.2017.12.006.
130. Zhang, H.; Kuai, X.; Ji, Z.; Li, Z.; Shi, R. Over-Expression of Small Ubiquitin-Related Modifier-1 and Sumoylated p53 in Colon Cancer. *Cell Biophys.* **2013**, *67*, 1081–1087, doi:10.1007/s12013-013-9612-x.
131. Zhang, J.; Huang, F.-F.; Wu, D.-S.; Li, W.-J.; Zhan, H.-E.; Peng, M.-Y.; Fang, P.; Cao, P.-F.; Zhang, M.-M.; Zeng, H.; et al. SUMOylation of insulin-like growth factor 1 receptor, promotes proliferation in acute myeloid leukemia. *Cancer Lett.* **2015**, *357*, 297–306, doi:10.1016/j.canlet.2014.11.052.
132. Zhang, P.; Li, H.; Yang, B.; Yang, F.; Zhang, L.-L.; Kong, Q.-Y.; Chen, X.-Y.; Wu, M.-L.; Liu, J. Biological significance and therapeutic implication of resveratrol-inhibited Wnt, Notch and STAT3 signaling in cervical cancer cells. *Genes Cancer* **2014**, *5*, 154–164, doi:10.18632/genesandcancer.15.
133. Zhang, P.; Yang, B.; Yao, Y.-Y.; Zhong, L.-X.; Chen, X.-Y.; Kong, Q.-Y.; Wu, M.-L.; Li, C.; Li, H.; Liu, J. PIAS3, SHP2 and SOCS3 Expression patterns in Cervical Cancers: Relevance with activation and resveratrol-caused inactivation of STAT3 signaling. *Gynecol. Oncol.* **2015**, *139*, 529–535, doi:10.1016/j.ygyno.2015.09.087.
134. Zhao, H.; Jiang, A.; Yu, M.; Bao, H. Identification of biomarkers correlated with diagnosis and prognosis of endometrial cancer using bioinformatics analysis. *J. Cell. Biochem.* **2020**, *121*, 4908–4921, doi:10.1002/jcb.29819.
135. Zhao, Z.; Tan, X.; Zhao, A.; Zhu, L.; Yin, B.; Yuan, J.; Qiang, B.; Peng, X. microRNA-214-mediated UBC9 expression in glioma. *BMB Rep.* **2012**, *45*, 641–646, doi:10.5483/bmbrep.2012.45.11.097.
136. Zhou, M.; Bian, Z.; Liu, B.; Zhang, Y.; Cao, Y.; Cui, K.; Sun, S.; Li, J.; Zhang, J.; Wang, X.; et al. Long noncoding RNA MCM3AP-AS1 enhances cell proliferation and metastasis in colorectal cancer by regulating miR-193a-5p/SENP1. *Cancer Med.* **2021**, *10*, 2470–2481, doi:10.1002/cam4.3830.
137. Zhou, Z.; Wang, M.; Li, J.; Xiao, M.; E Chin, Y.; Cheng, J.; Yeh, E.T.H.; Yang, J.; Yi, J. SUMOylation and SENP3 regulate STAT3 activation in head and neck cancer. *Oncogene* **2016**, *35*, 5826–5838, doi:10.1038/onc.2016.124.

138. Zhu, H.; Ren, S.; Bitler, B.; Aird, K.M.; Tu, Z.; Skordalakes, E.; Zhu, Y.; Yan, J.; Sun, Y.; Zhang, R. SPOP E3 Ubiquitin Ligase Adaptor Promotes Cellular Senescence by Degrading the SENP7 deSUMOylase. *Cell Rep.* **2015**, *13*, 1183–1193, doi:10.1016/j.celrep.2015.09.083.
139. Zhu, H.; Yu, J.J. Gene expression patterns in the histopathological classification of epithelial ovarian cancer. *Exp. Ther. Med.* **2010**, *1*, 187–192, doi:10.3892/etm_00000030.
